# Study on the Plateau Adaptive Synergistic Mechanism of Rumen Microbiome-Metabolome-Resistome in Tibetan Sheep

**DOI:** 10.3390/microorganisms13092049

**Published:** 2025-09-03

**Authors:** Xu Gao, Qianling Chen, Yuzhu Sha, Yanyu He, Xiu Liu, Xiaowei Chen, Pengyang Shao, Wei Huang, Yapeng He, Mingna Li, Zhiyun Hao, Bingang Shi, Jianfeng Xu

**Affiliations:** 1 College of Animal Science and Technology, Gansu Agricultural University, Lanzhou 730070, China; gx2049879994@163.com (X.G.); chenqianling223@163.com (Q.C.); 18894314093@163.com (Y.S.); cxw20002022@163.com (X.C.); shaopengyang666@163.com (P.S.); 18294737108@163.com (W.H.); 18894448066@163.com (Y.H.); limn@gsau.edu.cn (M.L.); haozy@st.gsau.edu.cn (Z.H.); shibg@st.gsau.edu.cn (B.S.); 2 School of Fundamental Sciences, Massey University, Palmerston North 4410, New Zealand; y.h@massey.ac.nz; 3 Gansu Institute of Animal Husbandry and Veterinary Medicine, Pingliang 744000, China

**Keywords:** tibetan sheep, Hu sheep, metagenome, metabolome, ARGs

## Abstract

Tibetan sheep are an important livestock breed adapted to the extreme environment of the Qinghai–Tibet Plateau (QTP). Their energy metabolism and environmental adaptability are highly dependent on the rumen microbiome. However, systematic comparisons of the rumen microbiome, its functions, and the resistome between plateau-adapted breeds and lowland breeds remain insufficient. In this study, 6 Tibetan sheep (TS) and 6 Hu sheep (HS) were selected. All the selected sheep had a body weight of 34 kg (±0.5 kg) and an age of 1 year (±1 month) and were all managed under local traditional natural grazing (without supplementary feeding). Using metagenomics and metabolomics techniques, systematic comparative analysis was conducted on the differences in rumen microbial community structure, functions, resistome, and metabolites between the two breeds. Metagenomic analysis showed that at the phylum level, the abundance of Bacteroidetes in the rumen of TS was significantly higher than that in HS (*p* < 0.05); at the genus level, the abundance of *Bacteroides* in TS was also significantly higher (*p* < 0.05). Carbohydrate-active enzymes (CAZy) analysis indicated that the abundance of Glycosyltransferases (GTs) and Carbohydrate-Binding Modules (CBMs) in the rumen of TS were significantly upregulated (*p* < 0.05), while HS was rich in various Glycoside Hydrolases (GHs). Comprehensive Antibiotic Resistance Database (CARD) analysis revealed that more than 60% of the Antibiotic Resistance Genes (ARGs) in the rumen of HS were present at higher levels than those in TS. Metabolomics identified a large number of differential metabolites, among which metabolites such as 2E,6Z,8Z,12E-hexadecatetraenoic acid, Leukotriene F4, and Tenurin were significantly upregulated in the rumen of TS. Correlation analysis showed that rumen microbial flora and their metabolites jointly act to regulate rumen ARGs. Specifically, microorganisms including Firmicutes and *Succiniclasticum* had a significantly positive correlation with ARGs such as *rpoB2* (*p* < 0.05), while differential metabolites like endomorphin-1 and Purothionin AII exhibited a significantly negative correlation with ARGs such as *rpoB2* (*p* < 0.05). Therefore, compared with HS, the synergistic effect of the rumen microbial flora, their metabolites, and the resistome in TS is an important characteristic and strategy for their adaptation to the hypoxic environment of the QTP, and also contributes to the formation of their unique rumen resistome. Despite being reared in the same plateau environment, the rumen microbiome of HS still retains low-altitude characteristics, which are manifested as high GHs activity and high ARGs abundance.

## 1. Introduction

The Qinghai–Tibet Plateau (QTP), known as the “Roof of the World” and the “Third Pole”, has an average altitude of 4500 m, making it the highest plateau in the world. It exhibits extreme ecological characteristics such as high cold and low oxygen; particularly under the cold environmental conditions, the average nighttime temperature is approximately −20 °C, and it usually drops below −30 °C in winter [1]. This unique environment has nurtured abundant endemic biological genetic resources, rendering it a natural laboratory for humans to study extreme environments. Tibetan sheep (*ovis aries*) are the main sheep breed on the Qinghai–Tibet Plateau. Formed through long-term natural and artificial selection, they are one of the three major coarse-wool sheep breeds in China. The stock size is about 30 million, accounting for more than 70% of the total stock of sheep on the QTP. It is not only the largest sheep population in China, but also a core component of the animal husbandry industry on the QTP. As the main source of livelihood for local herdsmen, Tibetan sheep (TS) provide meat, milk, wool, fuel and leather, and play an important role in maintaining Tibetan culture [2]. TS graze on natural pastures throughout the year without any supplementary feed [3]. Therefore, their carbohydrate and energy intake fluctuate significantly with the seasonal changes in forage supply. For instance, the vegetation growing period on the QTP lasts approximately 100 to 150 days, while the dormant period persists for around 7 months [4], It has typically harsh weather conditions. The grass germinates in early May, and the peak biomass occurs from late August to early September [5]. However, TS have been highly adapted to the harsh conditions of the QTP and can effectively cope with periods of energy scarcity [6], this is mainly due to the well-developed rumen system of ruminants.

As a signature organ of ruminants, the rumen harbors a complex and diverse microbial ecosystem that efficiently breaks down plant fibers [7]. This microbiome (the second genome) is closely related to the host’s nutrient absorption, environmental adaptability, and health status [8]. Studies have shown that the rumen resistome (a collection of resistance genes) is closely associated with the composition of the microbiome. For example, rumen Escherichia coli in the phylum Proteobacteria is the main carrier of antibiotic resistance genes (ARGs) in colostrum-fed goats, while *Prevotella ruminicolas* and *Fibrobacter succinogenes*, which are involved in cellulose degradation, become carriers of ARGs after the addition of starters [9]. ARGs in rumen bacteria are prone to spread to environments such as soil and water bodies through rumination and saliva, which may exert antibiotic selection pressure on environmental and human-related microbial communities [10]. Although existing studies have focused on the adaptability of rumen microbiota in plateau ruminants such as yaks [11,12]. However, as a representative livestock species on the QTP, systematic research is still lacking on how the rumen microbiome of TS mediates energy metabolism adaptation through structural remodeling and functional regulation, as well as the transmission mechanism of ARGs in the plateau pasture ecosystem.

The metabolic activity of the gastrointestinal microbiota can be affected by multiple factors such as the host itself, the host’s environment, and the microbiota [13]. Among the numerous influencing factors, the host’s genetic background is particularly crucial for shaping the structure of the rumen microbial community. Existing studies have shown that significant differences exist in the structure of the rumen microbial community among sheep breeds with different genetic backgrounds [14], However, geographical origin (via factors such as climate and altitude) and diet (pasture composition) are also key forces driving microbiome variation [15,16]. Hu sheep (HS) originated from the low-lying and humid Taihu Lake Basin in China. They are renowned for their high fecundity and have been widely introduced to various parts of the country to improve the reproductive performance of sheep [17]. Although HS have relatively high productivity, they face physiological challenges under the conditions of the plateau pastures-such as hypoxic environment, high altitude, intense ultraviolet radiation, low precipitation, and insufficient forage supply, factors which lead to a decline in their production performance. Therefore, by comparing HS with the native plateau breed TS to identify the differences between them, we aim to reveal the reasons why HS cannot adapt to the plateau environment, provide basic data for the introduction and improvement of HS, and explore whether HS can be better adapted to the plateau environment through breed improvement, thereby meeting the production needs of plateau areas.

In recent years, high-throughput metagenomic technologies have been widely applied to analyze the functions of uncultured bacteria in the gut microbiomes of different species and the ARGs they carry [18]. In addition, integrating multi-omics data such as gastrointestinal metagenomics and metabolomics to reveal the mechanisms related to host biological indicators (e.g., production performance) has become a research hotspot [19]. Metagenomic sequencing has been successfully used to analyze the rumen microbial taxonomy and functions of ordinary cattle [20], yaks [21] (revealing that their rumen microbes encode a more abundant gene pool of carbohydrate-active enzymes), and dairy cows [22] (discovering their unique rumen microbiome and drug resistance characteristics). Studies have also shown that the combined effect of the rumen microbiome and metabolome in dairy goats during early life affects their lifelong growth and lactation performance [23]. The rumen microbiome, metabolome, and host serum metabolome collectively determine the individual production performance of dairy cows [24]; However, despite the acclimatization of TS to the plateau environment, there remains a scarcity of systematic research on how their rumen microbiota mediates energy metabolism adaptation through microbial community structure remodeling and functional adjustment, as well as the transmission mechanism of ARGs in the alpine pasture ecosystem. Therefore, under the same altitude, feeding and management conditions, this study takes HS, a low-altitude introduced breed that has shown a certain adaptability to the alpine and hypoxic environment of the Qinghai–Tibet Plateau, as the control. It aims to reveal the interaction relationships among the rumen microbiome, metabolome, and resistome of TS at the multi-omics level, as well as their microecological mechanisms for synergistic adaptation to the alpine and hypoxic environment, and to evaluate the potential environmental risks of ARGs carried by the introduced HS breed.

## 2. Materials and Methods

### 2.1. Experimental Animals and Sample Collection

The experimental samples were collected from a herd owned by a local herdsman in Hezuo City, Gannan Tibetan Autonomous Prefecture, Gansu Province, China (103° E longitude, 35° N latitude). This area has an altitude of 3000 m, features a high-altitude humid climate, and has a relatively fragile ecological environment.

In this experiment, 6 representative TS and 6 representative HS were randomly selected from a large population. All selected sheep had a body weight of 34 kg (±0.5 kg) and an age of 1 year (±1 month). The experimental sheep showed normal feeding and rumination behaviors, had glossy wool, good body condition, and produced oval-shaped feces that adhered to each other after falling to the ground—all of which are characteristics of healthy sheep. All sheep were managed under the local traditional natural grazing system, with their forage mainly consisting of Poa poophagorum Bor, plants of the Poaceae family, Carex coninux, Argentina anserina, and Geranium platyanthum Duthie. No supplementary feeding was provided during the experiment. Detailed information on the nutritional components of the forage is shown in Table S1.

All animal experiments were conducted in strict accordance with the provisions of the Regulations on the Administration of Laboratory Animals (Ministry of Science and Technology of the People’s Republic of China, revised June 2004). The sample collection protocol has been approved by the Animal Husbandry and Management Committee of Gansu Agricultural University, with the approval number: GAU-LC-2020-27.

In this study, 6 TS and 6 HS were slaughtered separately for the collection of rumen contents. After slaughter by exsanguination via the jugular vein, the rumen was isolated immediately. From the ventral sac of the rumen of each sheep, 3 tubes of contents (using 5 mL cryopreservation tubes) were collected. After being placed into the cryopreservation tubes, the samples were immediately transferred to a pre-prepared liquid nitrogen tank for cryopreservation. Subsequently, the samples were transported back to the laboratory and stored at −80 °C for subsequent analysis.

### 2.2. Metagenomic Sequencing

#### 2.2.1. Library Construction and Sequencing

Sequencing libraries (n = 12) had been constructed with the NEBNext^®^ Ultra™ DNA Library Prep Kit for Illumina^®^. The specific steps were as follows: 1 μg of total DNA was taken, and the DNA was fragmented to 300–500 bp using an ultrasonic disruptor (Covaris, Woburn, MA, USA). End repair and A-tailing: The ends of DNA fragments were repaired to blunt ends using end repair enzymes, and an A-tail was added to the 3’ end. Adapter ligation: Illumina sequencing adapters (containing sample-specific index sequences) were ligated. PCR amplification: DNA fragments with ligated adapters were subjected to 8–10 cycles of PCR amplification using high-fidelity DNA polymerase (Phusion, NEB, Ipswich, MA, USA)) to enrich the library. Library purification: PCR products were purified using AMPure XP beads (Beckman Coulter, Brea, CA, USA) to remove unligated adapters and short-fragment DNA. Library quality control: The fragment size distribution of the library was analyzed using an Agilent Bioanalyzer 2100 (Agilent Technologies, Santa Clara, CA, USA), and the library concentration was quantified by qPCR. Library pooling and sequencing: Qualified libraries were pooled at equimolar concentrations and subjected to paired-end sequencing (PE150) on the Illumina NovaSeq 6000 platform (Illumina, Inc., San Diego, CA, USA).

#### 2.2.2. Data Quality Control and Bioinformatics Analysis

The raw reads obtained from sequencing contain low-quality data. To ensure the reliability of subsequent information analysis, it is necessary to filter them. In this study, the fastp0.23.2 software [25] was used to perform quality control and filtering on the raw tags to obtain high-quality sequencing data (clean tags). Bowtie2 [26] was used to align the sequencing data with the host reference genome (if provided) to remove host-derived contaminating sequences. Subsequently, the MEGAHIT1.2.9 software [27] was employed to perform metagenomic assembly on the filtered data, and contigs shorter than 300 bp were filtered out. Finally, the QUAST5.2.0 software was used to evaluate the assembly results. Gene prediction of the assembled genome sequences was performed using MetaGeneMark software (v3.26; http://exon.gatech.edu/meta_gmhmmp.cgi, accessed on 10 March 2024) [28] with its default parameters. Subsequently, the MMseqs2 software (v12-113e3; https://github.com/soedinglab/mmseqs2, accessed on 15 March 2024) [29] was used for redundant clustering of protein sequences, with the sequence identity cutoff set at 90% and the coverage cutoff set at 80%. Functional characterization of the assembled metagenomic sequences was conducted, and the databases used included: KEGG [30], CAZy [31], and CARD [32]. Using the species annotation data obtained by aligning non-repetitive gene sequences to the Nr database, the species makeup and their relative abundances of samples at each taxonomic level (kingdom, phylum, class, order, family, genus, and species) were calculated. On this basis, Alpha diversity analysis (calculating Ace, Chao1, Shannon, and Simpson indices to evaluate species richness and evenness) and Beta diversity analysis were conducted: PCoA [33], was used to visualize the inter-group differences in species composition and functional composition; ANOSIM was applied to assess the significance of these inter-group differences; and parametric tests (such as *t*-test) and non-parametric tests were performed on the inter-group differences in species composition and functional genes, respectively. Finally, to explore inter-group differential biomarkers, LEfSe analysis [34] was adopted for the rumen microbiota composition of TS and HS to identify statistically significant marker species.

### 2.3. Analysis of Rumen Microbial Metabolome

Metabolomic analysis of rumen fluid samples from Tibetan and HS (n = 12) was conducted using a liquid Chromatograph Mass Spectrometer (LC-MS) platform according to the method described by Chen [35]. After the samples were thawed at room temperature, 100 μL of each sample was measured out, and 500 μL of extraction solution (methanol: acetonitrile = 1:1 by volume) containing internal standards (1000:2, with an internal standard concentration of 2 mg/L) was added. The mixture was vortexed for 30 s, followed by ultrasonic treatment in an ice-water bath for 10 min, and then allowed to stand at −20 °C for 1 h. Subsequently, centrifugation was performed at 12,000 rpm and 4 °C for 15 min. Then, 500 μL of the supernatant was transferred to an EP tube, and the extract was dried in a vacuum concentrator. 150 μL of extraction solution (acetonitrile: water = 1:1 by volume) was added to the dried metabolites for reconstitution. The mixture was vortexed for another 30 s, subjected to ultrasonic treatment in an ice-water bath for 10 min, and then centrifuged at 12,000 rpm at 4 °C for 15 min. Finally, 120 μL of the supernatant was transferred to a 2 mL injection vial, and 10 μL was taken from each sample to be mixed into a QC sample for on-machine detection. Finally, 120 μL of the supernatant was transferred to a 2 mL injection vial, and 10 μL was taken from each sample to be mixed into a QC sample for on-machine detection. Samples were eluted using mobile phases in positive ion mode (ESI+) and negative ion mode (ESI−). The mobile phases consisted of solvent A (water and 5% acetonitrile with 0.1% formic acid) and solvent B (acetonitrile with 0.1%). The flow rate was 0.35 mL/min (note: there is a duplicate mention of flow rate; the latter “400 μL/min” is presumably a typo). The subsequent elution gradient of mobile phases (A:B) was as follows: 98%:2% during 0–0.25 min, 2%:98% during 10.0–13.0 min, and 98%:2% during 13.1–15.0 min. The ion source temperature was 150 °C, and the desolvation gas temperature was 500 °C. The flow rates of the purge gas and desolvation gas are 50 L/h and 800 L/h, respectively. The raw data collected by MassLynx (V4.2) were processed using Progenesis QI2.0 software. Based on the sample types, metabolite identification was performed by Progenesis QI2.0 software using online databases such as METLIN and the self-built database of Biomarker Technologies. The flow rates of the purge gas and desolvation gas are 50 L/h and 800 L/h, respectively. The raw data collected by MassLynx (V4.2) were processed using Progenesis QI2.0 software. Based on Progenesis QI2.0 software, metabolite identification was performed using online databases such as METLIN and the self-built database of Beijing Biomarker Technologies Co., Ltd. (Beijing, China). according to the sample types. The screening criteria for differential metabolites were FC > 1, *p* < 0.05, and VIP > 1. Moreover, KEGG functional annotation and enrichment analysis were conducted on the differential metabolites.

### 2.4. Metagenome-Metabolome Association Analysis

The integrated analysis of metagenomics and metabolomics was performed with reference to the method of McHardy et al. [36]. All species abundances were standardized. For correlation calculation, the maximum–minimum normalization method was first used to process the metabolites, species abundances, and microbial functional gene abundances. Then, the Pearson method was adopted to conduct correlation analysis between the abundances of differential metabolites and differential species/microbial functional genes, and a heatmap was drawn. Based on the correlation, the relationships between metabolites and microbial species could be obtained. The results of the association analysis between metabolites and microbial functional genes were integrated (|r| > 0.8, *p* < 0.05) to construct a multi-level regulatory relationship network of species-functional genes–metabolites. Meanwhile, the main contributing species of functional genes and their corresponding relationships were clarified based on metagenomic analysis. Sankey diagrams were used to show hierarchical flow relationships, and network diagrams were employed to present the overall interaction network. The results of correlation analysis were filtered based on the absolute value of the correlation coefficient (|CC| > 0.80) and the significance level (*p* < 0.05). From these, data subsets containing at least one metabolite cluster–microbe association pair ranked among the top 30 in terms of the absolute value of the correlation coefficient (sorted by |CC| in descending order) were retained for the drawing of correlation chord diagrams. All identified differential microbial genes and differential metabolites were co-mapped to the KEGG functional database to identify the functionally enriched pathways shared by them, and to clarify the main metabolic pathways and signal transduction pathways that both are involved in.

### 2.5. Data Analysis

The experimental data were initially arranged using Excel 2016. For correlation analysis, the Spearman correlation test method was uniformly adopted, with the screening criterion set as *p* < 0.05.

## 3. Results

### 3.1. Analysis of Rumen Microbial Composition and Function in Tibetan Sheep and Hu Sheep

**Figure 1:**
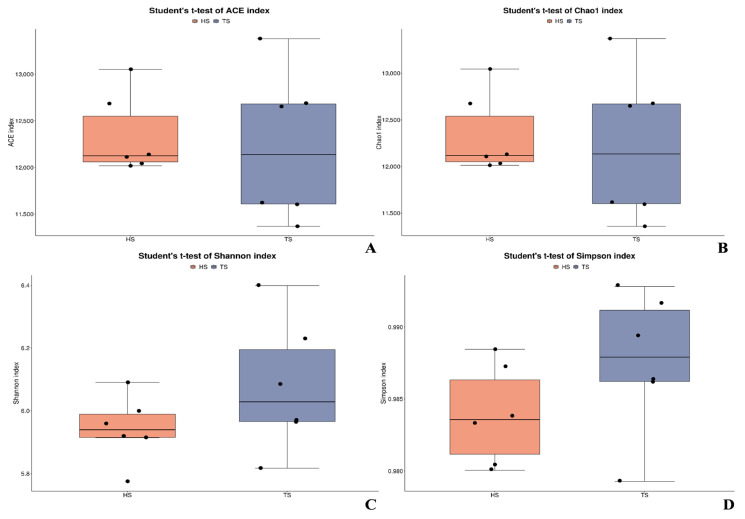
Microbial diversity analysis of Tibetan sheep and Hu sheep. (**A**–**D**) Microbial diversity indices (Chao1, ACE, Simpson, Shannon). Note: TS: Tibetan sheep HS: Hu sheep.

After excluding low-quality reads and those containing N bases, the average number of clean base pairs in the metagenomes of rumen fluid from the TS group and HS group was 10,928,238,869 bp and 11,240,291,310 bp, respectively. The HS group had 312,052,441 more base pairs than the TS group, representing a relative difference of 2.84%. After further removing the host genome sequences, the final number of valid reads in the two groups was 69,797,028.33 and 72,435,689.67, respectively. The HS group had 2,638,661.34 more valid reads than the TS group, with a relative difference of 3.78%. The Q20 and Q30 values were >97% and >95%, respectively (Table S2). These results indicated that the sequencing data were reliable and could be used for subsequent bioinformatics research. The average completeness of rumen microbial genome assembly in the TS group was 75.32%, while that in the HS group was 62.82%. (Table S3), the relative difference in TS compared to HS was 19.90%. Principal Coordinate Analysis (PCoA) revealed that the 6 individuals from populations at the same altitude clearly clustered together. This indicates that despite minor differences among individuals, there were breed-specific differences in the rumen microbial community structure between TS and HS. Furthermore, the HS microbial community showed a higher degree of aggregation, suggesting that its rumen microbial community is more conserved (Figure S1). According to the Venn diagram, there are 829,812 shared non-redundant genes between TS and HS, while TS and HS have 4,931,099 and 4,746,276 unique genes, respectively (Figure S2). Further analysis of the species composition of TS and HS revealed that the Chao1, ACE, Simpson, and Shannon indices (Figure 1A–D) of TS were all higher than those of HS, but the differences were not significant (*p* > 0.05).

**Figure 2:**
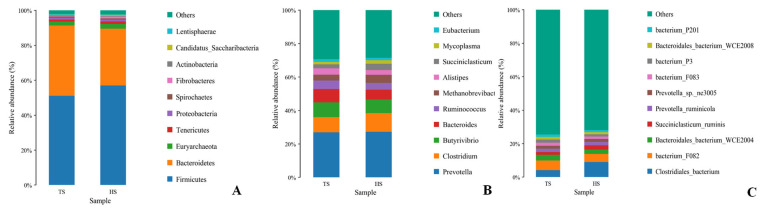
Species composition. (**A**) Bar chart of species composition at the phylum level. (**B**) Bar chart of species composition at the genus level. (**C**) Bar chart of species composition at the species level.

Analysis of microbial community composition revealed that at the phylum level, the core dominant bacterial phyla in both groups were Firmicutes (TS: 37.61%, HS: 41.34%) and Bacteroidota (TS: 29.41%, HS: 23.59%). Notably, Bacteroidota was significantly enriched in TS (*p* < 0.05) (Figure 2A). while the Firmicutes/Bacteroidetes ratio (F/B ratio) of HS (F/B: 1.75) was higher than that of TS (F/B: 1.28) (Figure S3). At the genus level, the dominant genera were *Prevotella* (TS: 11.57%, HS: 10.69%) and Clostridium (TS: 3.86%, HS: 4.37%), with no significant difference between the two groups. The next most abundant genera were *Bacteroides* (TS: 3.39%, HS: 2.29%) and *Butyrivibrio* (TS: 3.65%, HS: 3.18%). Notably, the relative abundance of *Bacteroides* in TS was significantly higher than that in HS (*p* < 0.05) (Figure 2B). The rank sum test showed that *Succiniclasticum* was extremely significantly enriched in HS (*p* < 0.001) (Figure S4). At the species level, the proportion of *Clostridiales_bacterium* was higher in HS, while the contents of bacterium_F082, *Prevotella_ruminicola*, and *Bacteroidales_bacterium_*WCE2004 were higher in TS (Figure 2C). LEfSe analysis identified species-level biomarkers, with the characteristic biomarker of TS being *Selenomonas ruminantium*, and the biomarkers of HS being Mycoplasma sp_CAG_877 and *Succiniclasticum ruminis* (Figures S5 and S6).

**Figure 3:**
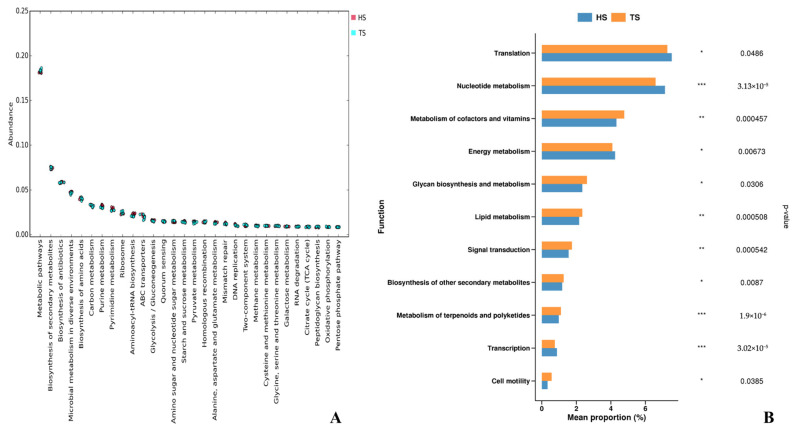
KEGG functional analysis. (**A**) Scatter plot of KEGG functional composition. (**B**) Bar chart of KEGG functional difference analysis. Note: * *p* < 0.05, ** *p* < 0.01, *** *p* < 0.001.

Functional analysis of rumen microorganisms based on KEGG pathways (Figure 3) showed that the KEGG functional genes of TS and HS were mainly enriched in pathways such as Metabolic pathways, Biosynthesis of secondary metabolites, Microbial metabolism in diverse environments, Biosynthesis of amino acids, Carbon metabolism, and Purine metabolism (Figure 3A). Further comparison of functional differences revealed that pathways such as Metabolism of cofactors and vitamins, Glycan biosynthesis and metabolism, and Lipid metabolism were significantly upregulated in TS (*p* < 0.05). However, the expression levels of the Translation, Nucleotide metabolism, and Energy metabolism pathways in HS were significantly higher than those in TS (*p* < 0.05) (Figure 3B).

**Figure 4:**
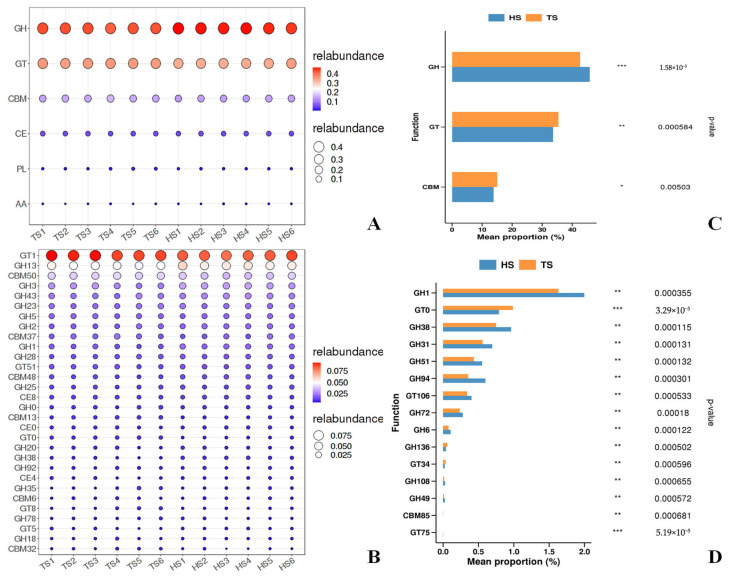
CAZy functional analysis. (**A**) CAZy content graph at the class level. (**B**) CAZy content graph at the family level. (**C**) Bar chart of differential analysis at the category level. (**D**) Bar chart of differential analysis at the family level. Note: * *p* < 0.05, ** *p* < 0.01, *** *p* < 0.001.

The results of carbohydrate-active enzyme analysis based on the CAZy database are shown in Figure 4. In terms of enzyme category composition (Figure 4A), the rumen microorganisms of TS and HS were jointly annotated into 6 categories of CAZymes, namely GHs, GTs, CBMs, Carbohydrate Esterases (CEs), Polysaccharide Lyases (PLs), and Auxiliary Activities (AAs). Among them, GHs and GTs are the most abundant. Analysis of the abundance of these enzyme families (Figure 4B) revealed that the abundance of GT1 was the highest, followed by GH13, CBM50, GH3, and CBM43. Analysis of differences in CAZy categories (Figure 4C) showed that the content of GHs in HS was extremely significantly higher than that in TS (*p* < 0.001), the content of GTs in TS was significantly higher than that in HS (*p* < 0.01), and the content of CBMs in TS was significantly higher than that in HS (*p* < 0.05). Differential analysis of these enzyme families (Figure 4D) indicated that the abundance of GT0 in TS was extremely significantly upregulated (*p* < 0.001), In the rumen microbes of HS, multiple GH families were highly significantly enriched, specifically including the GH1, GH38, GH31, and GH51 families (*p* < 0.01). This reflects the specialized differences between the two groups in the functions of carbohydrate degradation and synthesis.

### 3.2. Analysis of Rumen ARGs in Tibetan Sheep and Hu Sheep

**Figure 5:**
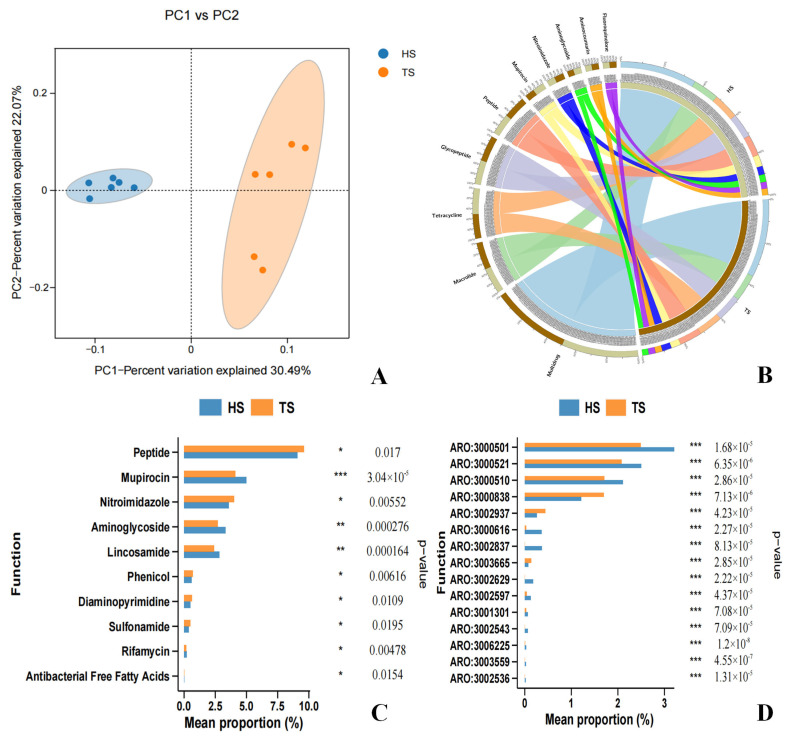
CARD functional analysis. (**A**) CARD principal coordinate analysis (PCoA) plot. (**B**) CARD antibiotic resistance gene composition diagram. (**C**) Bar chart of CARD difference analysis. (**D**) Bar chart of differential analysis at the ARO level. Note: * *p* < 0.05, ** *p* < 0.01, *** *p* < 0.001.

In this study, 486 and 502 types of ARGs with an abundance of ≥10 were detected in the rumen of the TS group and HS group, respectively (Table S4). PCoA revealed that the rumen resistome profiles of the TS group and HS group were clearly separated. Moreover, the sample points of the HS group’s resistome were tightly clustered, showing the characteristic of a highly conserved ARG composition. This suggests that breed difference is a key factor driving the differentiation of rumen resistomes (Figure 5A). Analysis of the composition categories of Comprehensive Antibiotic Resistance Database (CARD) functional genes (Figure 5B) showed that the most abundant category of CARD functional genes in both groups was Multidrug, followed by Macrolide, Tetracycline, Glycopeptide, and Peptide. Further heatmap clustering analysis revealed that the rumen resistomes of the TS group and HS group were clearly divided into two independent clusters, among which the expression levels of more than 60% of the ARGs in the HS group were higher than those in the TS group (Figure S7). Analysis of differences in CARD functional genes (Figure 5C) showed that Peptide had the highest abundance in both groups, with that in TS being significantly higher than in HS (*p* < 0.05). Similarly, the abundance of Nitroimidazole in TS was significantly higher than that in HS (*p* < 0.05). In contrast, there was an extremely significant difference in Mupirocin between the two groups (*p* < 0.001), with the abundance in TS being lower than that in HS. Indicating that there are significant differences in the resistome characteristics of the rumen microbiome between TS and HS. In addition, several linezolid resistance genes (*cfrA*, *cfr* (*C*), *cfr* (*B*), etc.) were identified in TS and HS (Table S4), As clinically important resistance genes, the detection of such genes in the rumen of plateau-grazing sheep requires attention to the potential risk of environmental transmission.

A rank-sum test was performed on the ARGs of TS and HS. After sorting the *p*-values in descending order, the top 15 ARGs were selected and then ranked by their abundance from high to low. The abundances of these ARGs showed significant differences between the two groups, among which 80% of the ARGs had extremely significantly higher abundances in HS than in TS (*p* < 0.001) (Figure 5D), These ARGs include *rpoB2* (ARO: 3000501), *Staphylococcus aureus mupA conferring resistance to mupirocin* (ARO: 3000521), *Staphylococcus aureus mupB conferring resistance to mupirocin* (ARO: 3000510), etc. More than 50% of the above-mentioned significantly different ARGs exert their resistance mechanism through antibiotic inactivation, including *lnuC*, *ANT (6)-Ib*, *AAC (6′)-Ie-APH (2″)-Ia*, etc. The resistance mechanisms of *mupA*, *mupB*, *RlmA(II)*, and the *vanS* gene in the *vanG* cluster are antibiotic target alteration. The resistance mechanisms of *rpoB2*, *mel*, and *arlR* are antibiotic target alteration, antibiotic target replacement, antibiotic target protection, and antibiotic efflux, respectively. These ARGs collectively mediate resistance to peptide antibiotics, rifamycin antibiotics, mupirocin, disinfecting agents, and antiseptics, etc. (Table S5).

**Figure 6:**
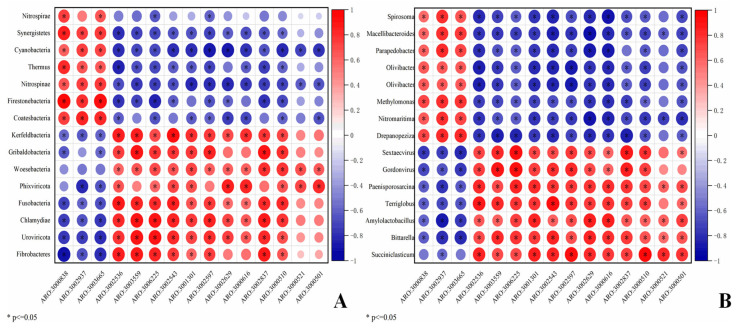
Correlation analysis between ARGs and species. (**A**) Heatmap of correlation analysis between species at the phylum level and ARGs. (**B**) Heatmap of correlation analysis between species at the genus level and ARGs.

To further clarify the relationship between ARGs and the microbial community in the rumen of TS and HS, the following steps were conducted: first, the rank sum test was performed on rumen microorganisms at the phylum and genus levels, respectively; then, after sorting by *p*-value in descending order, the top 15 were selected and re-sorted by abundance in descending order. The results showed that the screened microorganisms at the phylum and genus levels exhibited significant inter-group differences between TS and HS (Figures S8 and S9). A correlation analysis was conducted between the aforementioned significantly different phylum-level species and ARGs, revealing that phyla such as Synergistetes, Cyanobacteria, and Deinococcus_Thermus had a significant positive correlation (*p* < 0.05) with ARGs including *arlR* (ARO:3000838) and *NmcR* (ARO:3003665), while they were negatively correlated with ARGs such as *rpoB2* (ARO:3000501), *mupA* (ARO:3000521), *mupB* (ARO:3000510), *lnuC* (ARO:3002837), and *mel* (ARO:3000616) (Figure 6A). A correlation analysis between the significantly different genus-level species and ARGs revealed that genera such as *Spirosoma*, *Macellibacteroides*, and *Parapedobacter* exhibited significant positive correlations (*p* < 0.05) with ARGs including *arlR* (ARO:3000838) and *NmcR* (ARO:3003665), while they were negatively correlated with ARGs such as *rpoB2* (ARO:3000501), *mupA* (ARO:3000521), *mupB* (ARO:3000510), *lnuC* (ARO:3002837), and *mel* (ARO:3000616) (Figure 6B). Additionally, the analysis indicated that apart from the aforementioned microbial communities and ARGs, the remaining microbial communities were positively correlated with ARGs, and their abundances were higher in HS than in TS.

### 3.3. Analysis of Rumen Metabolome in Tibetan Sheep and Hu Sheep

**Figure 7:**
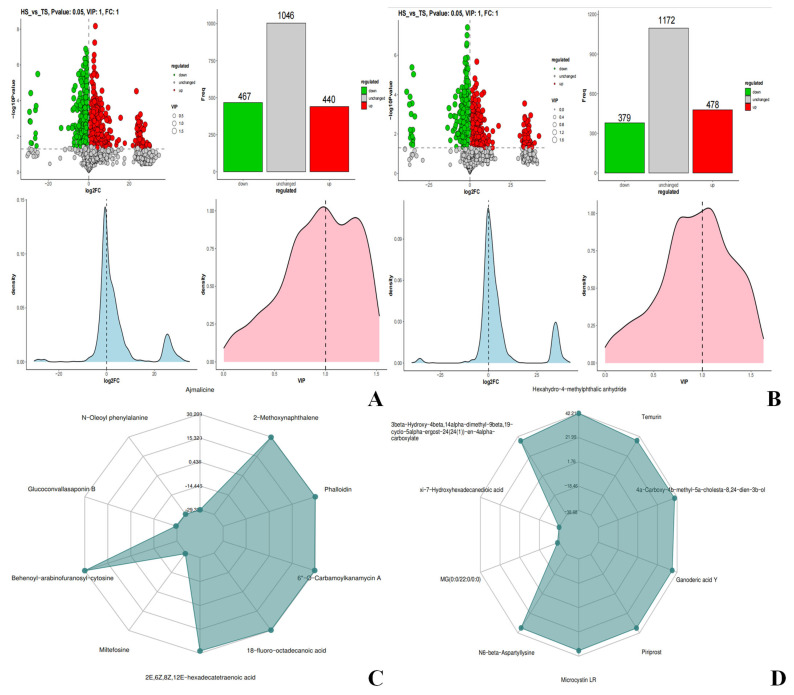
Description of rumen metabolome data quality control and differential metabolite analysis charts in TS and HS. (**A**,**B**) Statistical charts of differential metabolites. (**C**,**D**) Radar charts. ((**A**,**C**) are in positive ion mode. (**B**,**D**) are in negative ion mode).

Metabolomic qualitative and quantitative analyses were conducted on 12 samples from two sheep breeds, namely TS and HS. A total of 4004 peaks were detected in the positive ion mode (ESI+), among which 1953 metabolites were annotated (Table S6). In the negative ion mode (ESI−), a total of 4320 peaks were detected, among which 2029 metabolites were annotated (Table S7). Principal component analysis revealed that there were certain differences in rumen metabolites between the two groups under both positive and negative ion modes (Figures S10 and S11). The results of Spearman correlation coefficient analysis (Figures S12 and S13) showed that the two groups of samples had good detection repeatability and high data reliability. Meanwhile, it was found that TS and HS at the same altitude exhibited obvious aggregation characteristics in rumen metabolites during the analysis, and this aggregation pattern was species-specific. This further confirms the regulatory effect of breeds on rumen metabolism. Using fold change FC = 1, *p*-value = 0.05, and VIP = 1 as screening criteria, differential metabolite analysis was performed. In the positive ion mode, a total of 907 differential metabolites were identified, among which 440 were upregulated (Figure 7A). In the negative ion mode, a total of 857 differential metabolites were identified, with 478 being upregulated (Figure 7B). To further identify the key differential metabolites, the expression ratios were calculated based on the quantitative values of the differential metabolites. Subsequently, the top 10 metabolites with the largest absolute values of log_2_FC were selected to generate radar charts for visual display. In the positive ion mode, the upregulated metabolites included 2-Methoxynaphthalene, Phalloidin, 6″-O-Carbamoylkanamycin A, 18-fluoro-octadecanoic acid, 2E,6Z,8Z,12E-hexadecatetraenoic acid, and Behenoyl-arabinofuranosyl-cytosine, while the downregulated metabolites were Ajmalicine, N-Oleoyl phenylalanine, Glucoconvallasaponin B, and Miltefosine (Figure 7C). In the negative ion mode, the upregulated metabolites were Hexahydro-4-methylphthalic anhydrid, Tenurin, 4a-Carboxy-4b-methyl-5a-cholesta-8,24-dien-3b-ol, Ganoderic acid Y, Priprost, Microcystin LR, N6-beta-Aspartyllysine, and 3beta-Hydroxy-4beta,14alpha-dimethyl-9beta,19-cyclo-5alpha-ergost-24(241)-en-11alpha-carboxylate, and the downregulated metabolites were xi-7-Hydroxyhexadecanedioic acid and MG(0:0/22:0/0:0) (Figure 7D).

**Figure 8:**
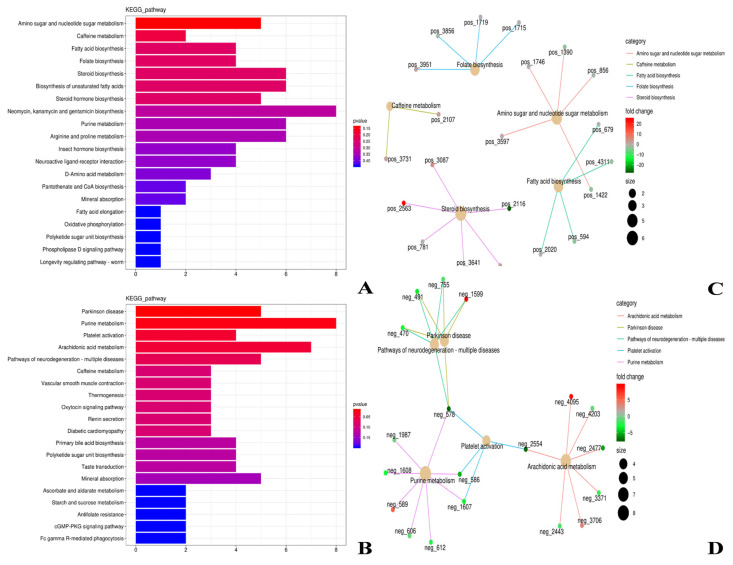
KEGG functional annotation and enrichment analysis of differential metabolites between TS and HS. (**A**,**B**) Enrichment bar charts. (**C**,**D**) Enrichment network plots. ((**A**,**C**): positive ion mode; (**B**,**D**): negative ion mode).

The results of KEGG functional annotation and enrichment analysis of differential metabolites in the rumen of TS and HS are shown in Figure 8. In the positive ion mode, the differential metabolites between TS and HS were mainly enriched in pathways such as Amino sugar and nucleotide sugar metabolism, Caffeine metabolism, and Fatty acid biosynthesis (Figure 8A). In addition, pathways such as Steroid biosynthesis and Folate biosynthesis were also enriched to a certain extent. In the negative ion mode, the differential metabolites between TS and HS were mainly enriched in pathways such as Parkinson disease, Purine metabolism, and Platelet activation (Figure 8B). Additionally, pathways like Arachidonic acid metabolism and Pathways of neurodegeneration-multiple diseases were also enriched to a certain extent. Further analysis of the key metabolites in the above significantly enriched pathways revealed that in the positive ion mode, metabolites including Ergosta-5,7,22,24 (28)-tetraen-3beta-ol, Ergosterol, and D-Glucosamine were all upregulated in TS (Figure 8C). In the negative ion mode, metabolites such as Xanthylic acid, 1-Methyl-4-phenylpyridinium, and Leukotriene F4 were also upregulated in TS (Figure 8D).

### 3.4. Correlation Analysis

#### 3.4.1. Rumen Microbiota–Metabolite Interactions

**Figure 9:**
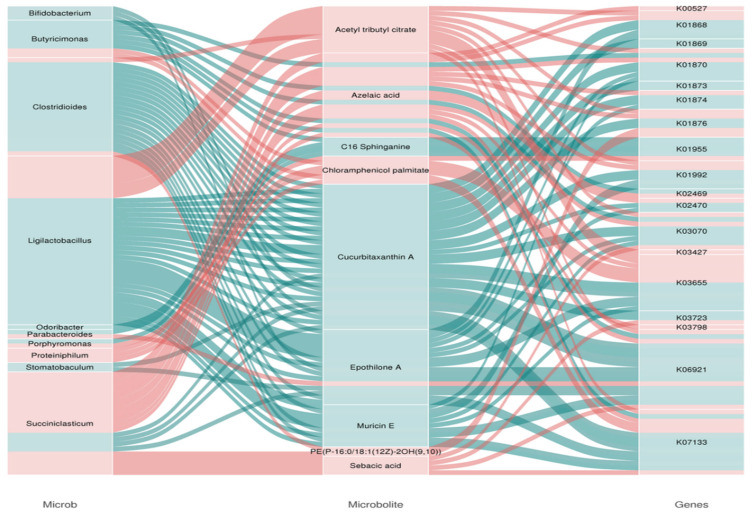
Sankey diagram of microbe-functional gene–metabolite association analysis.

Through the integrated analysis of metagenomic and metabolomic data, significant correlations (*p* < 0.05) were found among rumen metabolites, microbial communities, and the abundances of microbial functional genes (Figures S14 and S15). Additionally, a multi-level interaction network of rumen genera-functional genes–metabolites was constructed (Figure 9). *Ligilactobacillus*, *Clostridioides*, and the metabolite Acetyl tributyl citrate in TS and HS showed a significant positive correlation (*p* < 0.05) with the functions of threonyl-tRNA synthetase [EC:6.1.1.3] (K01868), preprotein translocase subunit SecA [EC:7.4.2.8] (K03070), isoleucyl-tRNA synthetase [EC:6.1.1.5] (K01870), ABC-2 type transport system permease protein (K01992), and aspartyl-tRNA synthetase (K01876). Microbial groups such as *Ligilactobacillus*, *Clostridioides*, *Succiniclasticum*, *Bifidobacterium*, and *Stomatobaculum*, as well as the metabolite Cucurbitaxanthin A, exhibited a significant negative correlation (*p* < 0.05) with functions including ABC-2 type transport system permease protein (K01992) and isoleucyl-tRNA synthetase [EC:6.1.1.5] (K01870). Further KEGG pathway enrichment analysis was performed on the differential metabolites and differential microbial functional genes to screen out the main biochemical and signal transduction pathways in which they were significantly involved together (Figure S16). The results showed that the differential metabolites and differential microorganisms were mainly enriched in functional pathways such as Glycerophospholipid metabolism, Glycolysis/Gluconeogenesis, Polyketide sugar unit biosynthesis, and Arachidonic acid metabolism.

#### 3.4.2. ARGs-Differential Metabolite Analysis

**Figure 10:**
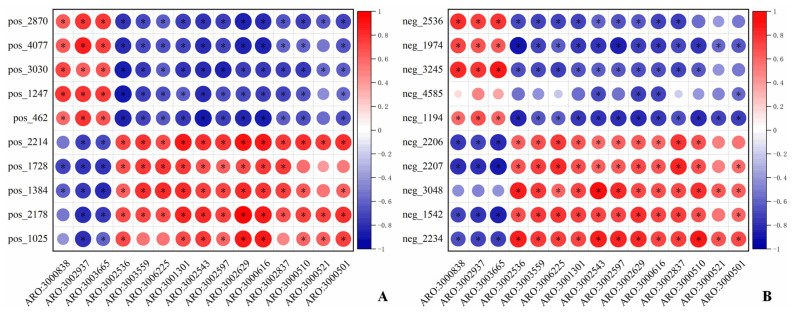
Heatmap of correlation analysis between differential metabolites and ARGs in positive and negative ion modes. (**A**) Positive ion mode. (**B**) Negative ion mode. Note: * *p* < 0.05.

To clarify the relationship between ARGs and differential metabolites in the rumen of TS and HS, correlation analysis was performed between the top 10 differential metabolites with the highest abundance in both positive and negative ion modes and ARGs, In positive ion mode, it was observed that metabolites such as Endomorphin-1 (pos_2870) and Purothionin AII (pos_4077) exhibited a significant positive correlation with ARGs including *NmcR* and *arlR*, while showing a negative correlation with ARGs like *rpoB2*. In contrast, metabolites such as Solutol HS 15 (pos_2214) and Amphibine B (pos_1025) were positively correlated with ARGs such as *rpoB2*, but negatively correlated with those like *NmcR* and *arlR* (*p* < 0.05) (Figure 10A). In negative ion mode, metabolites such as 2-Ethylsuberic acid (neg_2536) and 2,2′-Azobis(2-amidinopropane) (neg_1974) showed a positive correlation with *NmcR*, *arlR*, etc., while a negative correlation with ARGs like *rpoB2*. Metabolites including 4′-Hydroxy-5,7-dimethoxyflavan (neg_2206) and 8-Acetylegelolide (neg_2207) were positively correlated with ARGs such as *rpoB2*, but negatively correlated with ARGs like *NmcR* and *arlR* (*p* < 0.05) (Figure 10B).

## 4. Discussion

In this study, TS and HS raised under the same plateau conditions were selected, and metagenomics and metabolomics were used to compare the differences in rumen microorganisms and metabolites between them. The aims were to reveal the comprehensive characteristics of the rumen microbiome, explore the impact of breeds on the resistome as well as the potential relationship between the metabolome and the resistome, and finally clarify the potential mechanism of TS’s adaptation to the plateau environment through the interactions between rumen microorganisms and their metabolites. The composition and function of the rumen microbiome are regulated by various factors, including diet, host breed, and age [37]. Among these factors, the host genetic background (breed) plays a key role in driving the dynamic changes in the rumen microbiome [14]. Metagenomic analysis revealed that Firmicutes and Bacteroidetes are significantly enriched in the rumen of both TS and HS, which is consistent with previous studies [38]. This study found that the abundance of Firmicutes in the rumen microbiota of HS is higher, this phylum carries a variety of enzyme-encoding genes involved in energy metabolism, and its members can produce abundant digestive enzymes to decompose various substrates, thereby enhancing the host’s ability to digest and absorb nutrients [39,40], this indicates that HS have stronger digestive and absorptive capacities. The content of Bacteroidetes in the rumen of TS is significantly higher than that in HS. This bacterial phylum can decompose proteins and carbohydrates in forage to produce volatile fatty acids (VFAs) and promote rumen development [41,42]. Interestingly, in low-altitude ruminants and goats in semi-arid areas, the abundance of Bacteroidetes is lower than that of Firmicutes [43,44], This indicates that TS possess a stable energy utilization mechanism in the environment where forage supply is limited at high altitudes, which is crucial for maintaining the homeostasis of their rumen internal environment and adapting to harsh conditions. In addition, HS have a higher F/B ratio, which is associated with effective energy absorption and maintenance of metabolic balance in the body [45,46]. Studies have shown that the phylum Bacteroidetes is more inclined to produce propionic acid (a gluconeogenic precursor) [47], Therefore, the lower F/B ratio in TS—i.e., a higher proportion of the phylum Bacteroidetes—prioritizes the production of glucose precursors that can directly supply energy to the host. This difference may be associated with the evolution of a microbial community structure in TS that prioritizes efficient energy utilization during their long-term adaptation to the plateau. At the genus level, the relative abundance of Bacteroides in the rumen of TS is significantly higher (*p* < 0.05), and this genus can assist the host in digesting polysaccharides [48], and produce acetate, propionate, and succinate [49], indicating that TS possess a more robust energy conversion mechanism when facing the environment of scarce forage resources in alpine pastures. In addition, the high abundance of *Prevotella* in TS further supports their efficient energy acquisition strategy. This genus plays a role in the degradation and utilization of plant non-cellulosic polysaccharides, proteins, starch, and xylan [50], and can promote forage fermentation to generate high concentrations of VFAs [51]. At the species level, *bacterium_F082* and *Prevotella ruminicola* are the dominant species in TS. *Prevotella ruminicola* has been confirmed to be closely associated with cellulose degradation in ruminants after feeding, and can effectively utilize ammonia nitrogen and peptides to synthesize microbial proteins [9,52]. In addition, *bacterium_F082* has been reported to be associated with plant degradation [53]. These findings indicate that TS have strong nitrogen utilization efficiency and fiber decomposition capacity, which is related to their long-term survival in the environment with scarce forage. This study identified differential biomarkers in the rumen of ts and hs. Among these, *Selenomonas ruminantium*-a bacterium associated with fermenting carbohydrates, producing VFAs, maintaining rumen internal environment stability, and supporting animal health—exhibits a higher abundance in TS than in HS. [54,55]. This represents TS strategy for acquiring energy and maintaining their own homeostasis. In contrast, *Succiniclasticum ruminis*-the biomarker of HS-acts in the rumen by converting succinic acid into propionic acid. This process promotes energy utilization and nitrogen metabolism efficiency, regulates rumen fermentation [56], and thereby supports HS in maintaining their normal physiological needs. In addition, studies have shown that microorganisms can be used to identify the geographical origin and genetic identity of animals [15,16,57]. As a biomarker for TS adapting to the plateau environment, *Selenomonas ruminantium* can serve as a “microbial fingerprint” for identifying the geographical origin and genetic identity of the TS breed. Particularly in the large TS population-with an inventory of approximately 30 million in China-monitoring the composition of microbial biomarkers enables the assessment of the stability of the population’s genetic structure. Therefore, when TS and HS are raised under the same high-altitude feeding and management conditions, TS exhibit a higher abundance of Bacteroidetes, *Bacteroides*, *Prevotella*, *Prevotella ruminicola*, *bacterium_F082*, and *Selenomonas ruminantium* due to their inherent genetic factors. This constitutes the microbial mechanism underlying TS’s adaptation to alpine pastures, thereby endowing them with stronger digestive and absorptive capacities as well as higher energy metabolism efficiency to adapt to the plateau environment. Further analysis of KEGG functional differences revealed that the functions of TS in cofactor and vitamin metabolism, Glycan biosynthesis and metabolism, and Lipid metabolism were significantly enhanced, which could meet their energy requirements in the alpine and anoxic environment [58]. In contrast, the Translation, Nucleotide metabolism, and Energy metabolism of rumen microorganisms in HS were significantly higher than those in TS (*p* < 0.05), thus participating in various biological processes to ensure the normal growth and development of HS. The above analysis revealed that even under the same rearing environment, there are still significant differences in the enriched pathways of rumen microbes between TS and HS. This is mainly caused by breed differences-compared with HS, TS exhibit greater enrichment in pathways related to energy metabolism.

CAZymes are crucial for the survival and reproduction of microbial communities in carbohydrate-rich environments such as animal intestines, agricultural soils, forest soils, and marine sediments [59]. CAZymes are a class of enzymes capable of catalyzing the hydrolysis of glycosidic bonds in polysaccharides. The complexity of the structure of their substrates (polysaccharides) dictates that the degradation process requires the synergistic action of a diverse range of CAZymes. Currently, CAZymes can be divided into six categories: GHs, GTs, PLs, CEs, CBMs, and AAs [60]. In this study, the annotation of CAZymes in the rumen microbiota of TS and HS revealed that both breeds were enriched in the same functional categories such as GHs, GTs, and CBMs, indicating that the rumen microbiota has relatively stable core carbohydrate metabolic functions. However, there are significant breed differences in the expression levels of specific enzyme families. GHs are the core enzymes for rumen microorganisms to degrade plant biomass (such as cellulose, hemicellulose, and starch), responsible for catalyzing the hydrolysis of glycosidic bonds in complex carbohydrates [61]. The abundance of GHs in HS is significantly higher than that in TS, and multiple GH families (such as GH1, GH38, GH31, GH51, and GH94) are significantly enriched. This indicates that HS have a stronger ability to decompose cellulose, hemicellulose, and starch. GTs catalyze the activation of glycosyl groups and link them to specific acceptor molecules to form glycosidic bonds, playing a crucial role in carbohydrate synthesis as well as the adaptability and pathogenicity of host microorganisms [62]. In this study, the abundance of GTs was significantly upregulated in TS, which indicates that TS have stronger capabilities in the synthesis of carbohydrates such as glucose and greater adaptability of host-associated microbes. CBMs themselves lack catalytic activity; however, they can anchor CAZymes to the surface of substrates, thereby increasing contact time and surface area, and assisting hydrolases such as GHs and PLs in exerting their functions [31,63]. Therefore, the upregulated CBMs in the rumen of TS may play a crucial adaptive role in coping with high-altitude environmental stress by optimizing substrate binding and degradation efficiency. Thus, compared with HS, the differences in rumen microbes caused by genetic background enable TS to exhibit a distinct adaptive strategy—specifically, optimizing the functional efficiency of various enzymes through GTs and CBMs.

Studies have shown that antibiotics can disrupt the diversity of intestinal microorganisms and promote the spread of ARGs, thereby increasing the risk of diseases and the burden of drug resistance. This underscores the importance of strategies to protect the balance of the microecosystem [64]. Through CARD analysis, the most annotated antibiotic resistance types in both groups are peptides, tetracyclines, and macrolides. It is reported that the rumen microbiome contains a large number of ARGs. These genes may enter the intestines through saliva or rumen microorganisms, and then spread to humans through the environment such as soil and water [10]. In this study, 486 and 502 ARGs (with an abundance of 10 or higher) were detected in the rumen of TS and HS, respectively. This indicates that attention should be paid to the rumen resistome, especially those clinically important ARGs. Linezolid is one of the last-resort antimicrobial treatments in human clinical medicine and has not been approved for use in animal husbandry [65]. However, several linezolid resistance genes (*cfrA*, *cfr* (*C*), *cfr* (*B*), *cfr* (*D*), *optrA*, *poxtA*) were identified in the rumen samples of both groups in this study, which has also been confirmed in other studies [66,67]. Therefore, the high prevalence of clinically important ARGs in the rumen microbiota deserves great vigilance. The rumen microbiome serves as an important reservoir of ARGs, with the potential risk of transmission to humans and threat to public health [68]. Previous studies, as well as the present study, have confirmed that there is a strong correlation between the composition of rumen microorganisms and the resistome profile, indicating that changes in the structure of the microbial community directly affect the characteristics of the resistome [9], This is because the microbiome is the main carrier of ARGs [69], A correlation analysis between rumen microorganisms and ARGs revealed that microbial groups such as Fibrobacteres and *Succiniclasticum* were significantly positively correlated with ARGs including *rpoB2* (ARO: 3000501), *mupA* (ARO: 3000521), and *mupB* (ARO: 3000510). This supports the view that Fibrobacteres is a potential host of ARGs [70]. Both the above-mentioned microorganisms that are significantly positively correlated with ARGs and the associated ARGs themselves had significantly higher abundances in HS than in TS. This finding further confirms that rumen microorganisms are the main carriers of ARGs and reveals that introduced breeds may carry more resistance genes and pose a greater transmission risk. However, the specific molecular mechanisms behind these associations still need to be explored in depth. In summary, the rumen ARGs of TS are more conserved, and this difference is mainly shaped by breed. Compared with TS, the rumen ARGs of HS pose a more serious threat to the environment. Therefore, the microbial community structure selected by the host’s genetic background determines its functional output (antimicrobial resistance), and ultimately collectively shapes the host’s phenotype (antimicrobial resistance risk).

In addition, a correlation analysis was performed between ARGs and positive/negative ion metabolites in TS. It was found that in both positive and negative ion modes, the upregulated metabolites in TS and their positively correlated ARGs were also significantly upregulated, and the same was true for HS. This proves that certain metabolites have a synergistic relationship with ARGs. The upregulated metabolites in the positive ion mode of TS, namely Muricin E and 6-pentadecyl Salicylic Acid, can regulate and enhance host immunity [71,72], In the negative ion mode, 2,2′-Azobis (2-amidinopropane) can promote catalytic oxidation reactions and the production of anti-tumor metabolites [53]. Therefore, compared with HS, TS have a more robust immune mechanism when facing high-altitude environmental stress. They can produce more immune-related metabolites, which help TS maintain mucosal immune homeostasis without an intense inflammatory response, thereby avoiding energy consumption. At the same time, these metabolites can inhibit the abundance of ARGs such as *rpoB2*. This is a potential mechanism for the lower abundance of ARGs in TS than in HS, but the interaction between metabolites and ARGs requires further research.

As a key tool for analyzing the physiological and biochemical status of animals [24], metabolomics revealed in this study that there are significant differences in the rumen microbial metabolite profiles between TS and HS, and these differential metabolites may mediate different physiological adaptation processes. Under the positive ion mode, phalloidin, a metabolite upregulated in TS, can promote bile secretion [73]. 2E,6Z,8Z,12E-hexadecatetraenoic acid, which belongs to polyunsaturated fatty acids [74], may be involved in the regulation of cell membrane fluidity or signal transduction to adapt to the low-temperature environment of the plateau; Miltefosine has potential antifungal effects by disrupting fungal cell membranes and lipid metabolism [75]. The differential metabolites upregulated in TS under the positive ion mode play important roles in fatty acid metabolism, cell membrane adaptation, and intestinal barrier function of TS, which is an adaptive strategy for TS when facing environmental stress. Under the negative ion mode, Tenurin, which is upregulated in TS, has important functions in promoting neurite outgrowth, regulating cell communication, and maintaining energy balance, and may play a key role in the development and functional maintenance of the nervous system [76]. Owing to the physiological need of TS to adapt to the high-altitude hypoxic environment for a long time, Tenurin helps the host cope with the potential stress of the high-altitude environment on the neuromodulatory system. Priprost can inhibit leukotriene synthesis [77], which may increase lung blood flow and improve pulmonary vascular resistance in lambs. This finding is highly consistent with the host’s adaptive demand for respiratory function under the high-altitude hypoxic environment. Furthermore, the KEGG functional enrichment analysis of differential metabolites showed that under the positive ion mode, the differential metabolites between TS and HS were mainly enriched in metabolic pathways such as Amino sugar and nucleotide sugar metabolism, Caffeine metabolism, and Fatty acid biosynthesis. Among these, Amino sugar and nucleotide sugar metabolism was significantly upregulated in TS. Its key metabolite, D-Glucosamine, is a natural amino sugar that not only participates in bacterial cell wall construction and antibiotic synthesis but also has anti-inflammatory and antioxidant activities. It can enhance the host’s immune homeostasis by alleviating oxidative stress and inflammatory responses in the high-altitude environment [78]. Meanwhile, fatty acid metabolism-related pathways are more active in TS. This pathway not only affects cell membrane fluidity and energy storage but also participates in cell signal transduction as a precursor of second messengers, which can assist TS in coping with hypoxia and low-temperature stress from the perspectives of membrane adaptation and metabolic regulation [79,80]. In addition, Steroid biosynthesis plays a key role in maintaining cell structure, hormone production, lipid metabolism, and physiological regulation [81]. Under the negative ion mode, the differential metabolites between TS and HS were mainly enriched in pathways such as Purine metabolism and Arachidonic acid metabolism. Among these, Purine metabolism plays a core role in various life processes such as cellular energy transfer, nucleic acid synthesis, and signal transduction [82], and Arachidonic acid metabolism plays an important role in regulating inflammation, cell aging, and organ aging [83]. The expression of its enriched metabolites (e.g., Leukotriene F4) is upregulated in TS, which mainly plays a role in immune responses [84]. Both TS and HS participate in the above biological processes to ensure normal growth and development. In conclusion, there are significant differences in rumen microbial metabolites and their enriched pathways between TS and HS. Among them, a variety of metabolites related to energy absorption and immunity are upregulated in TS, indicating that TS have a strong immune capacity. This is another adaptive characteristic and strategy of TS when facing high-altitude environmental stress.

**Figure 11:**
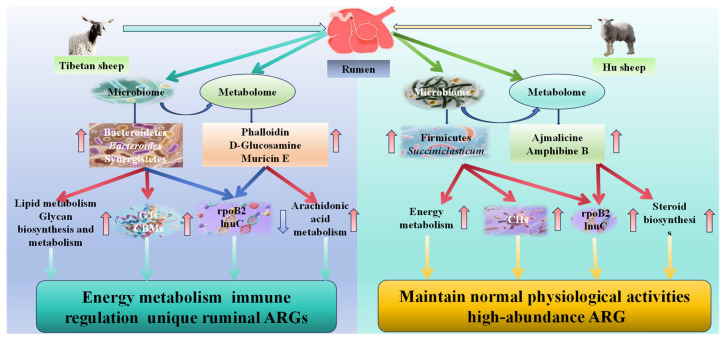
Host rumen microbiota–metabolite–ARG interaction model diagram.

In this study, a combined analysis of metabolomics and metagenomics was conducted, revealing that there is a significant correlation between these metabolites and rumen microorganisms as well as their microbial functions. Bacterial groups such as *Ligilactobacillus*, *Clostridioides*, *Succiniclasticum*, *Bifidobacterium*, and *Stomatobaculum*, as well as the metabolite Cucurbitaxanthin A, are significantly negatively correlated with functions such as ABC-2 type transport system permease protein (K01992) and isoleucyl-tRNA synthetase [EC:6.1.1.5] (K01870). *Bifidobacterium* can indirectly affect the function and metabolism of carotenoids by enhancing antioxidant capacity [85]. Cucurbitaxanthin A is a carotenoid unique to peppers, with pigmentation and antioxidant properties. The permease protein of the ABC-2 type transport system permease protein may be involved in the transmembrane transport and regulation of carotenoids, affecting their accumulation in cells and biosynthesis efficiency [85]. It indicates that there is a certain regulatory relationship between rumen microorganisms, their functions, and metabolites in TS and HS. Further comparison between TS and HS revealed that differential metabolites and differential metagenes were mainly enriched in Glycerophospholipid metabolism, Glycolysis/Gluconeogenesis, and Polyketide sugar unit biosynthesis. Among them, the Glycerophospholipid metabolism pathway may affect the adaptability of TS and HS in high-altitude environments, because lipid composition influences the fluidity and function of cell membranes, thereby affecting oxygen exchange, cold resistance, and energy metabolism [86]. The enrichment of Glycolysis/Gluconeogenesis plays an important role in energy production, metabolic regulation, and carbon source conversion, as well as their coordinated mechanisms [87,88], which are crucial for meeting the growth needs of TS and HS. Combined analysis showed that there is a significant correlation between rumen microorganisms and metabolites in TS and HS. Key metabolites and microbial functional genes are mainly enriched in pathways such as glycerophospholipid metabolism and glycolysis/gluconeogenesis. This indicates that the microbiome and the host have formed a high degree of synergy in energy metabolism, and these pathways are of great significance for energy metabolism and adaptation in the plateau environment.(Figure 11)

## 5. Conclusions

This study clarified through integrated multi-omics analysis that TS have developed a host genetics-dominated synergistic regulatory mechanism involving microbiota–metabolites–ARGs during their adaptation to the plateau. Specifically, microbes such as Bacteroidetes and *Bacteroides* are significantly enriched in their rumen, which promotes the optimization of GTs and CBMs enzyme systems to enhance fiber energy utilization. Meanwhile, they upregulate lipid metabolism and immunomodulatory metabolites, thereby strengthening the host’s adaptability and anti-inflammatory capacity. Additionally, they maintain rumen microecological homeostasis by inhibiting ARGs. These findings indicate that TS adopt a plateau adaptation strategy characterized by efficient energy utilization and drug resistance control through host-microbe interactions. In contrast, HS still retain lowland-type microbial functions and the risk of ARG transmission. This suggests that the shaping effect of host genetic background on the functional characteristics of the rumen microbial community is stronger than the impact of short-term plateau environmental exposure. Since HS still maintain the characteristics of the rumen resistome typical of low-altitude breeds, this alerts the animal husbandry industry to attach great importance to the prevalence of ARGs in the rumen microbiome (e.g., the risks posed by introduced breeds) and their potential risk of diffusion in the ecological environment.

## Data Availability

The raw sequencing data generated in this study have been deposited in the National Center for Biotechnology Information (NCBI) Sequence Read Archive (SRA) database under the BioProject accession number PRJNA1296747. We confirm that the present study was reported in strict accordance with the ARRIVE guidelines. All aspects of the experimental design, animal handling, data collection, and reporting were carried out in compliance with these guidelines to ensure transparency and reproducibility of the research.

## Supplementary Materials

The following supporting information can be downloaded at: https://www.mdpi.com/article/10.3390/microorganisms13092049/s1, Figure S1: Microbial diversity analysis of Tibetan sheep and Hu sheep. Venn diagram; Figure S2. Microbial diversity analysis of Tibetan sheep and Hu sheep. Principal Coordinate Analysis (PCoA) diagram; Figure S3. Species composition. Box plot of F/B ratio analysis; Figure S4. Species composition. Bar Chart of Differential Species Abundance Analysis at the Genus Level; Figure S5. Species composition. LDA analysis plot; Figure S6. Species composition. LDA analysis plot; Figure S7. CARD functional analysis. Heatmap of Differential Abundance at the ARO Level; Figure S8. Bar chart of differential analysis at the phylum level; Figure S9. Bar chart of differential analysis at the genus level; Figure S10. Description of rumen metabolome data quality control and differential metabolite analysis charts in TS and HS. 3D Principal Component Analysis (3D PCA). (are in positive ion mode.); Figure S11. Description of rumen metabolome data quality control and differential metabolite analysis charts in TS and HS. 3D Principal Component Analysis (3D PCA). (are in negative ion mode); Figure S12. Description of rumen metabolome data quality control and differential metabolite analysis charts in TS and HS. Replicate sample correlation analysis. (are in positive ion mode.); Figure S13. Description of rumen metabolome data quality control and differential metabolite analysis charts in TS and HS. Replicate sample correlation analysis. (are in negative ion mode); Figure S14. Integrated Analysis of Metagenomics and Metabolomics. Chord diagram of the correlation between metabolite clusters and microbial functions; Figure S15. Integrated Analysis of Metagenomics and Metabolomics. Chord diagram of the correlation between metabolite clusters and microbial species; Figure S16. Integrated Analysis of Metagenomics and Metabolomics. Scatter plot of KEGG pathway analysis for differential metabolites and differential microbial functional genes; Table S1. Nutrient composition of forage in Gannan; Table S2. Statistics of metagenomic sequencing data quality control for rumen fluid samples from TS and HS sheep breeds; Table S3. Completeness statistics of rumen microbial genome assembly in TS and HS sheep breeds; Table S4. Detection of ≥10–abundance ARGs in TS/HS rumen; Table S5. Detailed information of top 15 differential antibiotic resistance genes (ARGs) between TS and HS sheep breeds, including resistance mechanisms and mediated antimicrobial categories; Table S6. Summary of metabolite detection and annotation in rumen samples of TS and HS sheep breeds under positive ion mode (ESI+); Table S7. Summary of metabolite detection and annotation in rumen samples of TS and HS sheep breeds under positive ion mode (ESI−).

## References

[1] Li B., Jia G., Wen D., Zhao X., Zhang J., Xu Q., Zhao X., Jiang N., Liu Z., Wang Y. (2022). Rumen microbiota of indigenous and introduced ruminants and their adaptation to the qinghai-tibetan plateau. Front. Microbiol..

[2] Zhang Z., Xu D., Wang L., Hao J., Wang J., Zhou X., Wang W., Qiu Q., Huang X., Zhou J. (2016). Convergent evolution of rumen microbiomes in high-altitude mammals. Curr. Biol..

[3] Yang C., Gao P., Hou F., Yan T., Chang S., Chen X., Wang Z. (2018). Relationship between chemical composition of native forage and nutrient digestibility by tibetan sheep on the qinghai-tibetan plateau. J. Anim. Sci..

[4] Xue B., Zhao X.Q., Zhang Y.S. (2005). Seasonal changes in weight and body composition of yak grazing on alpine-meadow grassland in the qinghai-tibetan plateau of china. J. Anim. Sci..

[5] Xue D., Chen H., Zhao X., Xu S., Hu L., Xu T., Jiang L., Zhan W. (2017). Rumen prokaryotic communities of ruminants under different feeding paradigms on the qinghai-tibetan plateau. Syst. Appl. Microbiol..

[6] Sun Y., Angerer J., Hou F. (2015). Effects of grazing systems on herbage mass and liveweight gain of tibetan sheep in eastern qinghai-tibetan plateau, china. Rangel. J..

[7] Pan X., Cai Y., Li Z., Chen X., Heller R., Wang N., Wang Y., Zhao C., Wang Y., Xu H. (2021). Modes of genetic adaptations underlying functional innovations in the rumen. Sci. China Life Sci..

[8] Lv W., Liu X., Sha Y., Shi H., Wei H., Luo Y., Wang J., Li S., Hu J., Guo X. (2021). Rumen fermentation-microbiota-host gene expression interactions to reveal the adaptability of tibetan sheep in different periods. Animals.

[9] Chai J., Zhuang Y., Cui K., Bi Y., Zhang N. (2024). Metagenomics reveals the temporal dynamics of the rumen resistome and microbiome in goat kids. Microbiome.

[10] Auffret M.D., Dewhurst R.J., Duthie C., Rooke J.A., John Wallace R., Freeman T.C., Stewart R., Watson M., Roehe R. (2017). The rumen microbiome as a reservoir of antimicrobial resistance and pathogenicity genes is directly affected by diet in beef cattle. Microbiome.

[11] Qiu Q., Wang L., Wang K., Yang Y., Ma T., Wang Z., Zhang X., Ni Z., Hou F., Long R. (2015). Yak whole-genome resequencing reveals domestication signatures and prehistoric population expansions. Nat. Commun..

[12] Qiu Q., Zhang G., Ma T., Qian W., Wang J., Ye Z., Cao C., Hu Q., Kim J., Larkin D.M. (2012). The yak genome and adaptation to life at high altitude. Nat. Genet..

[13] Rowland I.R. (1988). Factors affecting metabolic activity of the intestinal microflora. Drug Metab. Rev..

[14] Douglas J., Worgan H.J., Easton G.L., Poret L., Wolf B.T., Edwards A., Davies E., Ross D., McEwan N.R. (2016). Microbial diversity in the digestive tract of two different breeds of sheep. J. Appl. Microbiol..

[15] Sun G., Zhang H., Wei Q., Zhao C., Yang X., Wu X., Xia T., Liu G., Zhang L., Gao Y. (2019). Comparative analyses of fecal microbiota in european mouflon (ovis orientalis musimon) and blue sheep (pseudois nayaur) living at low or high altitudes. Front. Microbiol..

[16] Guo R., Zhang W., Shen W., Zhang G., Xie T., Li L., Jinmei J., Liu Y., Kong F., Guo B. (2023). Analysis of gut microbiota in chinese donkey in different regions using metagenomic sequencing. BMC Genom..

[17] He H., Fang C., Liu L., Li M., Liu W. (2024). Environmental driving of adaptation mechanism on rumen microorganisms of sheep based on metagenomics and metabolomics data analysis. Int. J. Mol. Sci..

[18] Campbell T.P., Sun X., Patel V.H., Sanz C., Morgan D., Dantas G. (2020). The microbiome and resistome of chimpanzees, gorillas, and humans across host lifestyle and geography. ISME J..

[19] Wang Q., Wang K., Wu W., Giannoulatou E., Ho J.W.K., Li L. (2019). Host and microbiome multi-omics integration: Applications and methodologies. Biophys. Rev..

[20] Gao H., Yu Y., Lv Y., Wang D., Li H., Li Z., Zhang Y., Chen L., Leng J. (2023). Metagenomic sequencing reveals the taxonomic and functional characteristics of rumen micro-organisms in gayals. Microorganisms.

[21] Zhao C., Wang L., Ke S., Chen X., Kenéz Á., Xu W., Wang D., Zhang F., Li Y., Cui Z. (2022). Yak rumen microbiome elevates fiber degradation ability and alters rumen fermentation pattern to increase feed efficiency. Anim. Nutr..

[22] Sun H., Peng K., Xue M., Liu J. (2021). Metagenomics analysis revealed the distinctive ruminal microbiome and resistive profiles in dairy buffaloes. Anim. Microbiome.

[23] Wang D., Chen L., Tang G., Yu J., Chen J., Li Z., Cao Y., Lei X., Deng L., Wu S. (2023). Multi-omics revealed the long-term effect of ruminal keystone bacteria and the microbial metabolome on lactation performance in adult dairy goats. Microbiome.

[24] Xue M., Sun H., Wu X., Liu J., Guan L.L. (2020). Multi-omics reveals that the rumen microbiome and its metabolome together with the host metabolome contribute to individualized dairy cow performance. Microbiome.

[25] Chen S., Zhou Y., Chen Y., Gu J. (2018). Fastp: An ultra-fast all-in-one FASTQ preprocessor. Bioinformatics.

[26] Langmead B., Salzberg S.L. (2012). Fast gapped-read alignment with bowtie 2. Nat. Methods.

[27] Li D., Liu C., Luo R., Sadakane K., Lam T. (2015). MEGAHIT: An ultra-fast single-node solution for large and complex metagenomics assembly via succinct de bruijn graph. Bioinformatics.

[28] Zhu W., Lomsadze A., Borodovsky M. (2010). Ab initio gene identification in metagenomic sequences. Nucleic Acids Res..

[29] Steinegger M., Soding J. (2017). MMseqs2 enables sensitive protein sequence searching for the analysis of massive data sets. Nat. Biotechnol..

[30] Kanehisa M., Goto S., Kawashima S., Okuno Y., Hattori M. (2004). The KEGG resource for deciphering the genome. Nucleic Acids Res..

[31] Cantarel B.L., Coutinho P.M., Rancurel C., Bernard T., Lombard V., Henrissat B. (2009). The carbohydrate-active EnZymes database (CAZy): An expert resource for glycogenomics. Nucleic Acids Res..

[32] Jia B., Raphenya A.R., Alcock B., Waglechner N., Guo P., Tsang K.K., Lago B.A., Dave B.M., Pereira S., Sharma A.N. (2017). CARD 2017: Expansion and model-centric curation of the comprehensive antibiotic resistance database. Nucleic Acids Res..

[33] Sakaki T., Takeshima T., Tominaga M., Hashimoto H., Kawaguchi S. (1994). Recurrence of ICA-PCoA aneurysms after neck clipping. J. Neurosurg..

[34] Segata N., Izard J., Waldron L., Gevers D., Miropolsky L., Garrett W.S., Huttenhower C. (2011). Metagenomic biomarker discovery and explanation. Genome Biol..

[35] Chen Q., Sha Y., Liu X., He Y., Chen X., Yang W., Gao M., Huang W., Wang J., He J. (2024). Unique rumen micromorphology and microbiota-metabolite interactions: Features and strategies for tibetan sheep adaptation to the plateau. Front. Microbiol..

[36] McHardy I.H., Goudarzi M., Tong M., Ruegger P.M., Schwager E., Weger J.R., Graeber T.G., Sonnenburg J.L., Horvath S., Huttenhower C. (2013). Integrative analysis of the microbiome and metabolome of the human intestinal mucosal surface reveals exquisite inter-relationships. Microbiome.

[37] Furman O., Shenhav L., Sasson G., Kokou F., Honig H., Jacoby S., Hertz T., Cordero O.X., Halperin E., Mizrahi I. (2020). Stochasticity constrained by deterministic effects of diet and age drive rumen microbiome assembly dynamics. Nat. Commun..

[38] Zhang Y.K., Zhang X.X., Li F.D., Li C., Li G.Z., Zhang D.Y., Song Q.Z., Li X.L., Zhao Y., Wang W.M. (2021). Characterization of the rumen microbiota and its relationship with residual feed intake in sheep. Animal.

[39] Kaakoush N.O. (2015). Insights into the role of erysipelotrichaceae in the human host. Front. Cell. Infect. Microbiol..

[40] Nuriel-Ohayon M., Neuman H., Koren O. (2016). Microbial changes during pregnancy, birth, and infancy. Front. Microbiol..

[41] Xiang R., Oddy V.H., Archibald A.L., Vercoe P.E., Dalrymple B.P. (2016). Epithelial, metabolic and innate immunity transcriptomic signatures differentiating the rumen from other sheep and mammalian gastrointestinal tract tissues. PeerJ.

[42] Jami E., White B.A., Mizrahi I. (2014). Potential role of the bovine rumen microbiome in modulating milk composition and feed efficiency. PLoS ONE.

[43] Kim M., Morrison M., Yu Z. (2011). Status of the phylogenetic diversity census of ruminal microbiomes. FEMS Microbiol. Ecol..

[44] Cunha I.S., Barreto C.C., Costa O.Y.A., Bomfim M.A., Castro A.P., Kruger R.H., Quirino B.F. (2011). Bacteria and archaea community structure in the rumen microbiome of goats (capra hircus) from the semiarid region of brazil. Anaerobe.

[45] Murphy E.F., Cotter P.D., Healy S., Marques T.M., O’Sullivan O., Fouhy F., Clarke S.F., O’Toole P.W., Quigley E.M., Stanton C. (2010). Composition and energy harvesting capacity of the gut microbiota: Relationship to diet, obesity and time in mouse models. Gut.

[46] Fernando S.C., Purvis H.T.N., Najar F.Z., Sukharnikov L.O., Krehbiel C.R., Nagaraja T.G., Roe B.A., Desilva U. (2010). Rumen microbial population dynamics during adaptation to a high-grain diet. Appl. Environ. Microbiol..

[47] Kawasoe J., Uchida Y., Kawamoto H., Miyauchi T., Watanabe T., Saga K., Tanaka K., Ueda S., Terajima H., Taura K. (2022). Propionic acid, induced in gut by an inulin diet, suppresses inflammation and ameliorates liver ischemia and reperfusion injury in mice. Front. Immunol..

[48] Li Y., Hu X., Yang S., Zhou J., Zhang T., Qi L., Sun X., Fan M., Xu S., Cha M. (2017). Comparative analysis of the gut microbiota composition between captive and wild forest musk deer. Front. Microbiol..

[49] Koh A., De Vadder F., Kovatcheva-Datchary P., Bäckhed F. (2016). From dietary fiber to host physiology: Short-chain fatty acids as key bacterial metabolites. Cell.

[50] Liu H., Xu T., Xu S., Ma L., Han X., Wang X., Zhang X., Hu L., Zhao N., Chen Y. (2019). Effect of dietary concentrate to forage ratio on growth performance, rumen fermentation and bacterial diversity of tibetan sheep under barn feeding on the qinghai-tibetan plateau. PeerJ.

[51] Shi W., Moon C.D., Leahy S.C., Kang D., Froula J., Kittelmann S., Fan C., Deutsch S., Gagic D., Seedorf H. (2014). Methane yield phenotypes linked to differential gene expression in the sheep rumen microbiome. Genome Res..

[52] Purushe J., Fouts D.E., Morrison M., White B.A., Mackie R.I., Coutinho P.M., Henrissat B., Nelson K.E. (2010). Comparative genome analysis of prevotella ruminicola and prevotella bryantii: Insights into their environmental niche. Microb. Ecol..

[53] Teng Z., Zhang N., Zhang L., Zhang L., Liu S., Fu T., Wang Q., Gao T. (2024). Integrated multi-omics reveals new ruminal microbial features associated with peanut vine efficiency in dairy cattle. Life.

[54] Ricke S.C., Martin S.A., Nisbet D.J. (1996). Ecology, metabolism, and genetics of ruminal selenomonads. Crit. Rev. Microbiol..

[55] Sawanon S., Koike S., Kobayashi Y. (2011). Evidence for the possible involvement of selenomonas ruminantium in rumen fiber digestion. FEMS Microbiol. Lett..

[56] Li W., Ma T., Zhang N., Deng K., Diao Q. (2025). Dietary fat supplement affected energy and nitrogen metabolism efficiency and shifted rumen fermentation toward glucogenic propionate production via enrichment of succiniclasticum in male twin lambs. J. Integr. Agric..

[57] Alvanou M.V., Loukovitis D., Melfou K., Giantsis I.A. (2024). Utility of dairy microbiome as a tool for authentication and traceability. Open Life Sci..

[58] Jenkins T.C. (1993). Lipid metabolism in the rumen. J. Dairy Sci..

[59] Zheng J., Hu B., Zhang X., Ge Q., Yan Y., Akresi J., Piyush V., Huang L., Yin Y. (2023). DbCAN-seq update: CAZyme gene clusters and substrates in microbiomes. Nucleic Acids Res..

[60] Lombard V., Golaconda Ramulu H., Drula E., Coutinho P.M., Henrissat B. (2014). The carbohydrate-active enzymes database (CAZy) in 2013. Nucleic Acids Res..

[61] Brulc J.M., Antonopoulos D.A., Miller M.E.B., Wilson M.K., Yannarell A.C., Dinsdale E.A., Edwards R.E., Frank E.D., Emerson J.B., Wacklin P. (2009). Gene-centric metagenomics of the fiber-adherent bovine rumen microbiome reveals forage specific glycoside hydrolases. Proc. Natl. Acad. Sci. USA.

[62] Zhu F., Zhang H., Wu H. (2015). Glycosyltransferase-mediated sweet modification in oral streptococci. J. Dent. Res..

[63] Jones D.R., Thomas D., Alger N., Ghavidel A., Inglis G.D., Abbott D.W. (2018). SACCHARIS: An automated pipeline to streamline discovery of carbohydrate active enzyme activities within polyspecific families and de novo sequence datasets. Biotechnol. Biofuels.

[64] Fishbein S.R.S., Mahmud B., Dantas G. (2023). Antibiotic perturbations to the gut microbiome. Nat. Rev. Microbiol..

[65] Wang Y., Li X., Fu Y., Chen Y., Wang Y., Ye D., Wang C., Hu X., Zhou L., Du J. (2020). Association of florfenicol residues with the abundance of oxazolidinone resistance genes in livestock manures. J. Hazard. Mater..

[66] Zhao Q., Wang Y., Wang S., Wang Z., Du X., Jiang H., Xia X., Shen Z., Ding S., Wu C. (2016). Prevalence and abundance of florfenicol and linezolid resistance genes in soils adjacent to swine feedlots. Sci. Rep..

[67] Li J., Shao B., Shen J., Wang S., Wu Y. (2013). Occurrence of chloramphenicol-resistance genes as environmental pollutants from swine feedlots. Environ. Sci. Technol..

[68] Hitch T.C.A., Thomas B.J., Friedersdorff J.C.A., Ougham H., Creevey C.J. (2018). Deep sequence analysis reveals the ovine rumen as a reservoir of antibiotic resistance genes. Environ. Pollut..

[69] Li X., Stokholm J., Brejnrod A., Vestergaard G.A., Russel J., Trivedi U., Thorsen J., Gupta S., Hjelmsø M.H., Shah S.A. (2021). The infant gut resistome associates with e. Coli, environmental exposures, gut microbiome maturity, and asthma-associated bacterial composition. Cell Host Microbe.

[70] Cui G., Bhat S.A., Li W., Wei Y., Kui H., Fu X., Gui H., Wei C., Li F. (2019). Gut digestion of earthworms significantly attenuates cell-free and -associated antibiotic resistance genes in excess activated sludge by affecting bacterial profiles. Sci. Total Environ..

[71] Gnanaprakasam J.N.R., López-Bañuelos L., Vega L. (2021). Anacardic 6-pentadecyl salicylic acid induces apoptosis in breast cancer tumor cells, immunostimulation in the host and decreases blood toxic effects of taxol in an animal model. Toxicol. Appl. Pharmacol..

[72] Zorofchian Moghadamtousi S., Rouhollahi E., Karimian H., Fadaeinasab M., Firoozinia M., Ameen Abdulla M., Abdul Kadir H. (2015). The chemopotential effect of annona muricata leaves against azoxymethane-induced colonic aberrant crypt foci in rats and the apoptotic effect of acetogenin annomuricin e in HT-29 cells: A bioassay-guided approach. PLoS ONE.

[73] Rahman K., Coleman R. (1986). Selective biliary lipid secretion at low bile-salt-output rates in the isolated perfused rat liver. Effects of phalloidin. Biochem. J..

[74] 2e,4z,6z,8z-Decatetraenedioic Acid. https://pubchem.ncbi.nlm.nih.gov/compound/9543652.

[75] Spadari C.D.C., Borba-Santos L.P., Rozental S., Ishida K. (2023). Miltefosine repositioning: A review of potential alternative antifungal therapy. J. Mycol. Med..

[76] Dodsworth T.L., Lovejoy D.A. (2022). Role of teneurin c-terminal associated peptides (TCAP) on intercellular adhesion and communication. Front. Neurosci..

[77] Lebidois J., Soifer S.J., Clyman R.I., Heymann M.A. (1987). Piriprost: A putative leukotriene synthesis inhibitor increases pulmonary blood flow in fetal lambs. Pediatr. Res..

[78] Dalirfardouei R., Karimi G., Jamialahmadi K. (2016). Molecular mechanisms and biomedical applications of glucosamine as a potential multifunctional therapeutic agent. Life Sci..

[79] Günenc A.N., Graf B., Stark H., Chari A. (2022). Fatty acid synthase: Structure, function, and regulation. Sub-Cell. Biochem..

[80] Currie E., Schulze A., Zechner R., Walther T.C., Farese R.V.J. (2013). Cellular fatty acid metabolism and cancer. Cell Metab..

[81] Taves M.D., Donahue K.M., Bian J., Cam M.C., Ashwell J.D. (2023). Aire drives steroid hormone biosynthesis by medullary thymic epithelial cells. Sci. Immunol..

[82] Gordon M.P., Intrieri O.M., Brown G.B. (1957). On the metabolism of purine. J. Biol. Chem..

[83] Qian C., Wang Q., Qiao Y., Xu Z., Zhang L., Xiao H., Lin Z., Wu M., Xia W., Yang H. (2025). Arachidonic acid in aging: New roles for old players. J. Adv. Res..

[84] Melo C.F.O.R., Bachur L.F., Delafiori J., Dabaja M.Z., de Oliveira D.N., Guerreiro T.M., Tararam C.A., Busso-Lopes A.F., Moretti M.L., Catharino R.R. (2020). Does leukotriene f4 play a major role in the infection mechanism of candida sp.?. Microb. Pathog..

[85] Chen J., Chen X., Ho C.L. (2021). Recent development of probiotic bifidobacteria for treating human diseases. Front. Bioeng. Biotechnol..

[86] Farooqui A.A., Horrocks L.A., Farooqui T. (2000). Glycerophospholipids in brain: Their metabolism, incorporation into membranes, functions, and involvement in neurological disorders. Chem. Phys. Lipids.

[87] Schink S.J., Christodoulou D., Mukherjee A., Athaide E., Brunner V., Fuhrer T., Bradshaw G.A., Sauer U., Basan M. (2022). Glycolysis/gluconeogenesis specialization in microbes is driven by biochemical constraints of flux sensing. Mol. Syst. Biol..

[88] Li Z., Chen Y., Liu D., Zhao N., Cheng H., Ren H., Guo T., Niu H., Zhuang W., Wu J. (2015). Involvement of glycolysis/gluconeogenesis and signaling regulatory pathways in saccharomyces cerevisiae biofilms during fermentation. Front. Microbiol..

