# Supplementary material for: Study on the Plateau Adaptive Synergistic Mechanism of Rumen Microbiome-Metabolome-Resistome in Tibetan Sheep
Source: Microorganisms. 2025 Sep 3;13(9):2049. doi: 10.3390/microorganisms13092049 (PMC12472100; doi:10.3390/microorganisms13092049)
Supplement: Supplementary file 1 [file microorganisms-13-02049-s001.zip › microorganisms-3795397-supplementary/microorganisms-3795397.Figure S1-16.pdf]

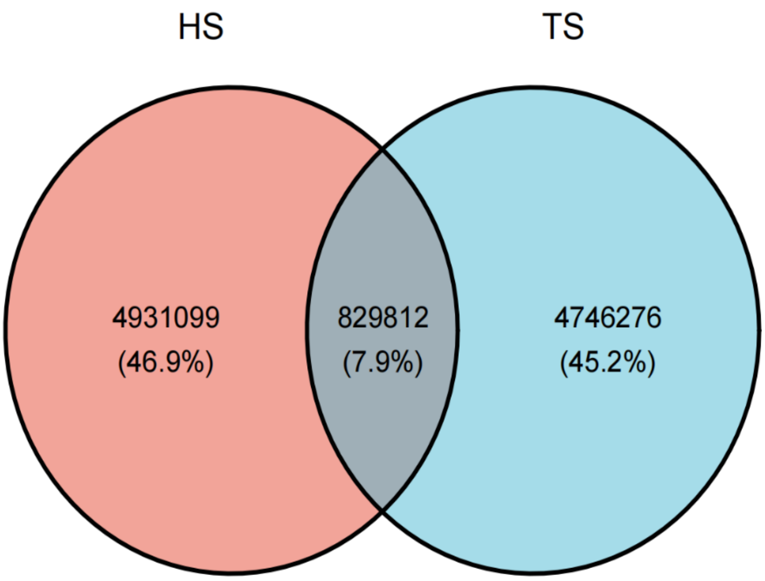

Figure S1. Microbial diversity analysis of Tibetan sheep and Hu sheep.Venn diagram.

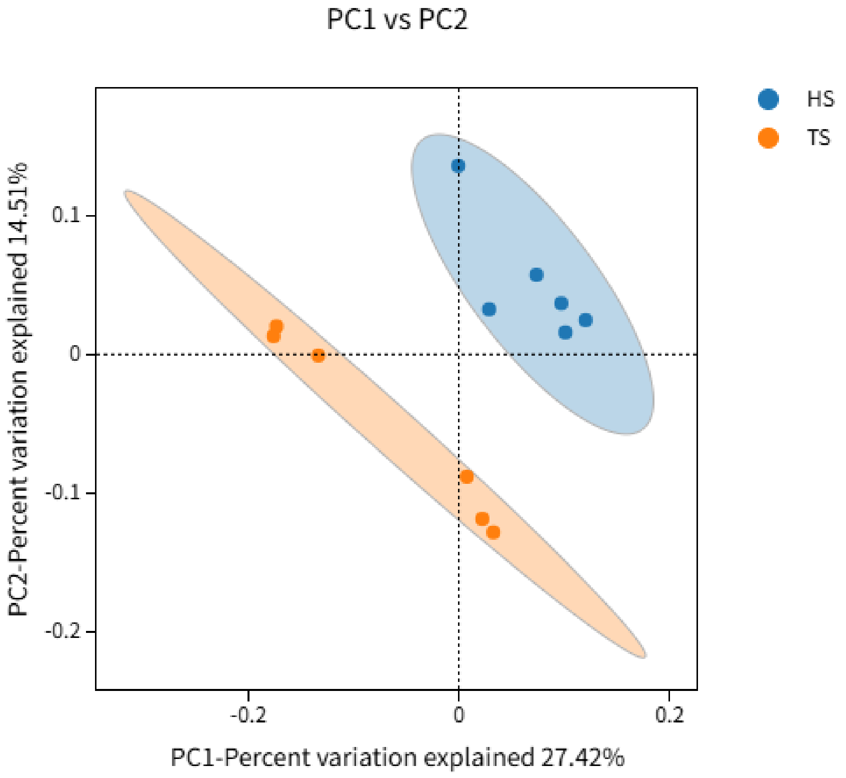

Figure S2. Microbial diversity analysis of Tibetan sheep and Hu sheep.Principal Coordinate Analysis (PCoA) diagram.

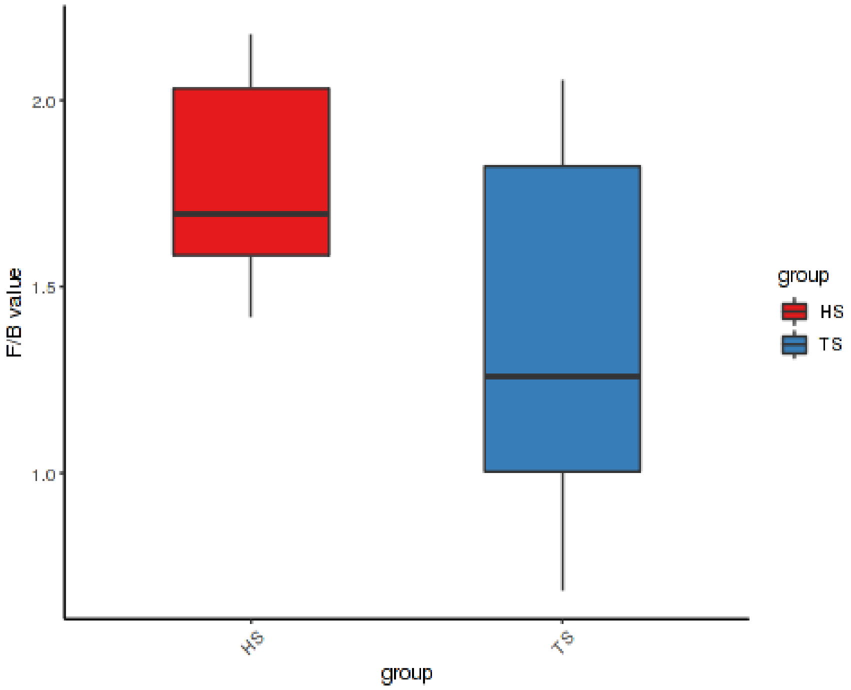

Figure S3. Species composition. Box plot of F/B ratio analysis.

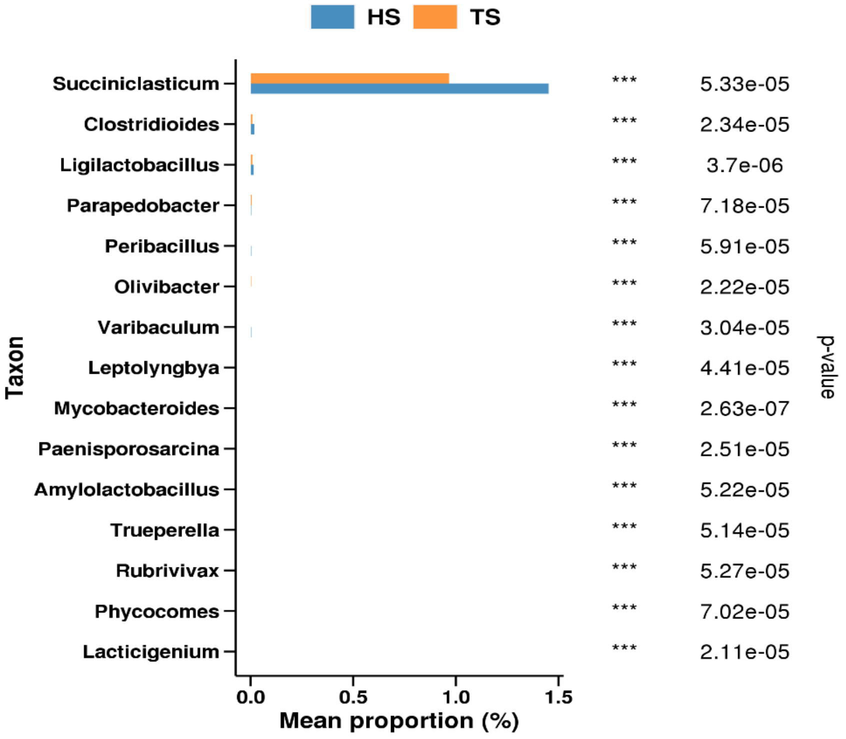

Figure S4. Species composition. Bar Chart of Differential Species Abundance Analysis at the Genus Level

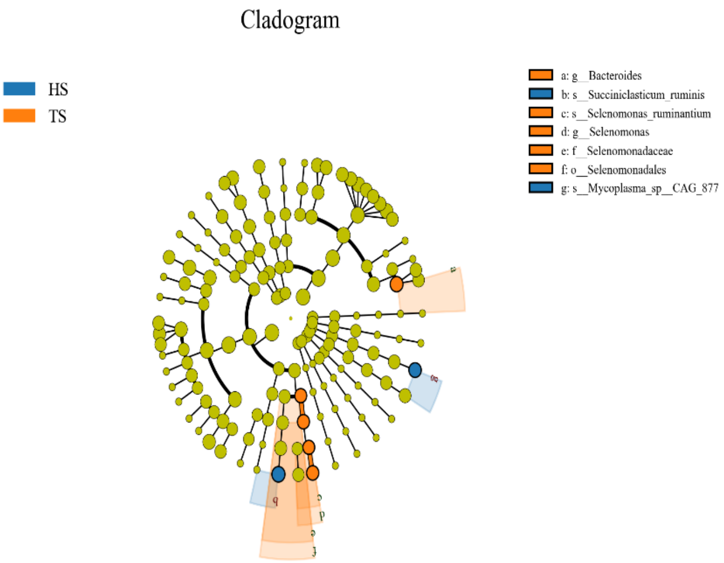

Figure S5.Species composition. LDA analysis plot.

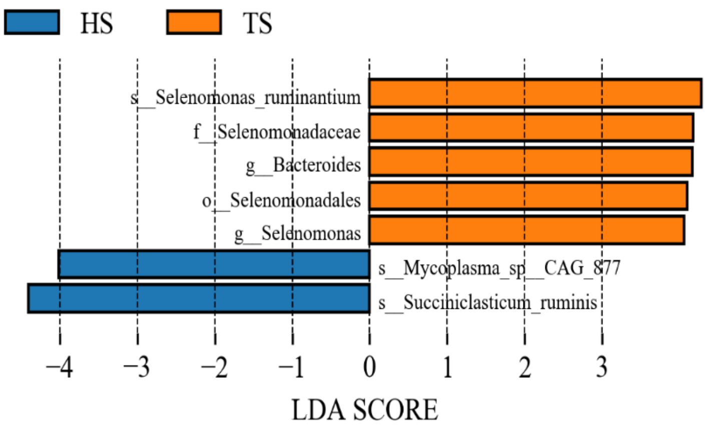

Figure S6.Species composition. LDA analysis plot.

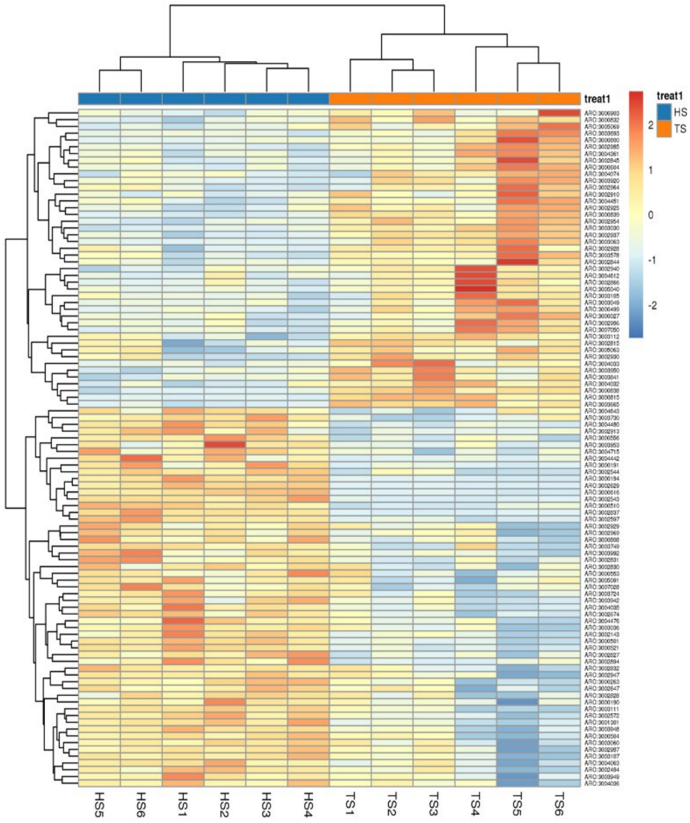

Figure S7.CARD functional analysis. Heatmap of Differential Abundance at the ARO Level.

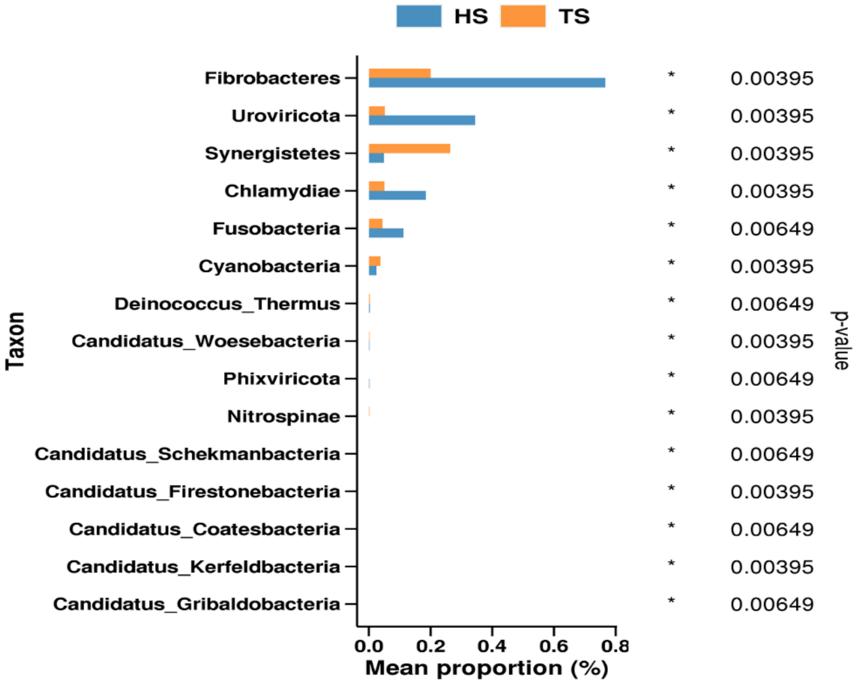

Figure S8. Bar chart of differential analysis at the phylum level.

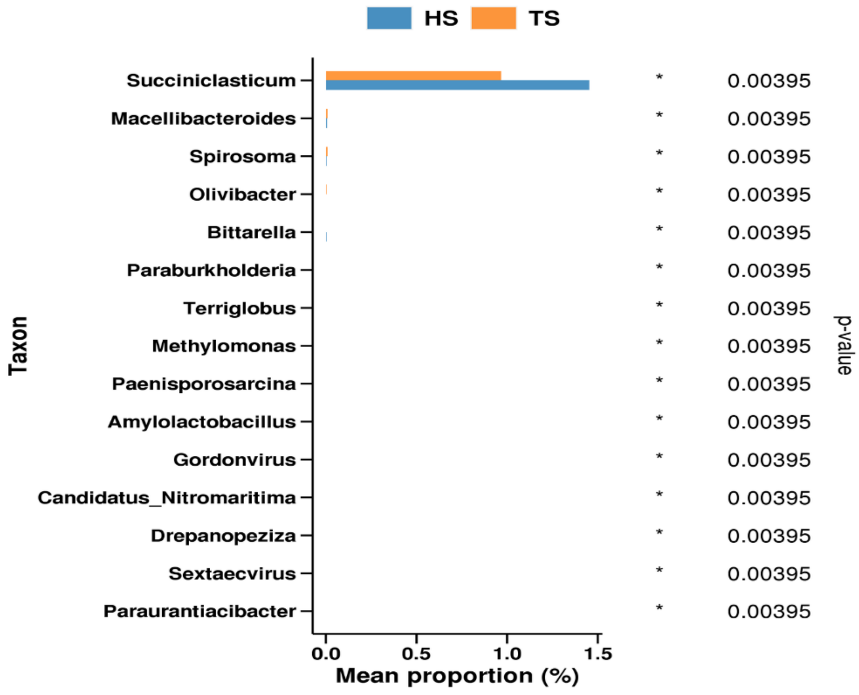

Figure S9.Bar chart of differential analysis at the genus level.

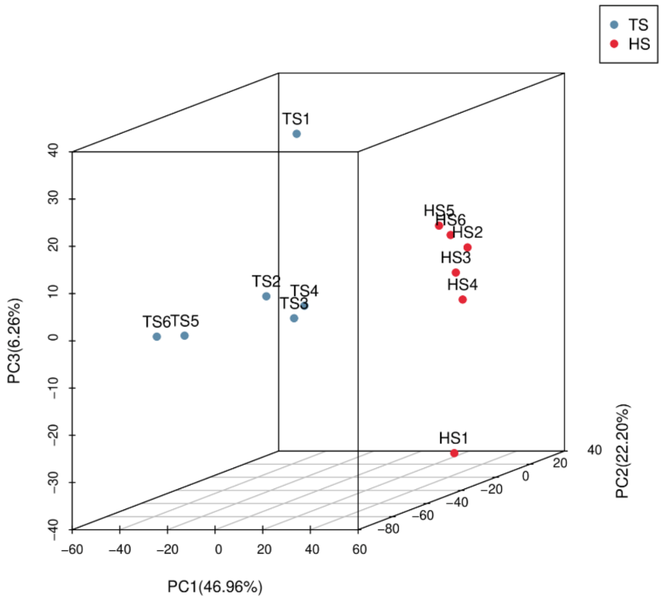

Figure S10. Description of rumen metabolome data quality control and differential metabolite analysis charts in TS and HS. 3D Principal Component Analysis (3D PCA). （are in positive ion mode. ）

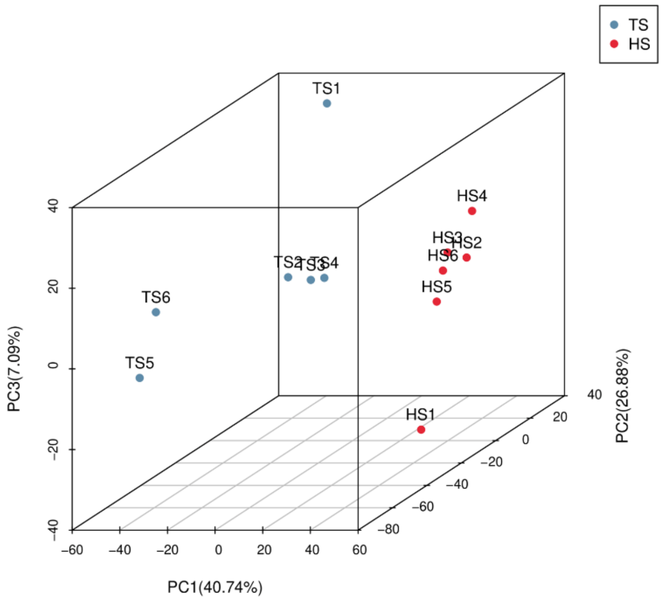

Figure S11. Description of rumen metabolome data quality control and differential metabolite analysis charts in TS and HS. 3D Principal Component Analysis (3D PCA). （are in negative ion mode）

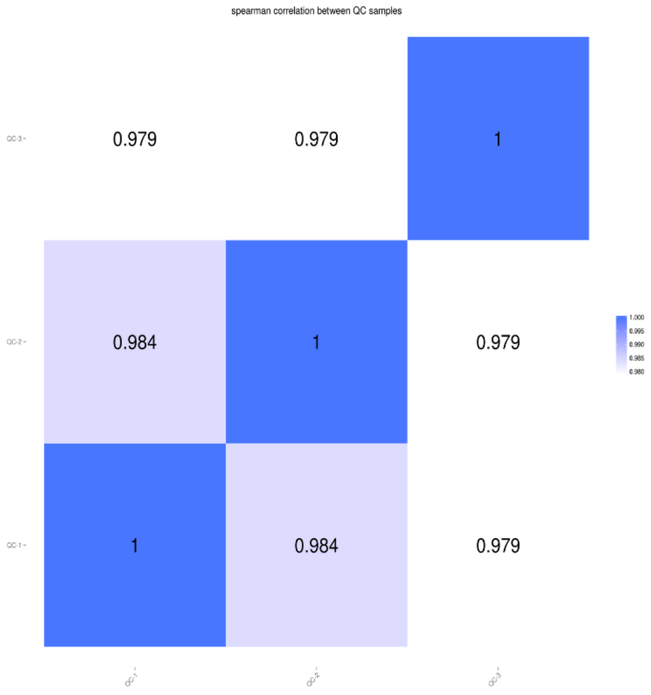

Figure S12. Description of rumen metabolome data quality control and differential metabolite analysis charts in TS and HS. Replicate sample correlation analysis. （are in positive ion mode.）

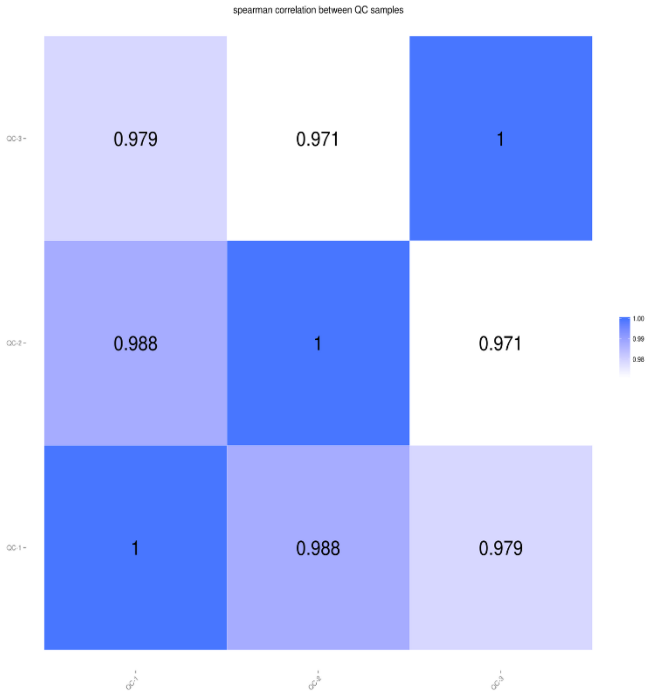

Figure S13. Description of rumen metabolome data quality control and differential metabolite analysis charts in TS and HS. Replicate sample correlation analysis. （are in negative ion mode）

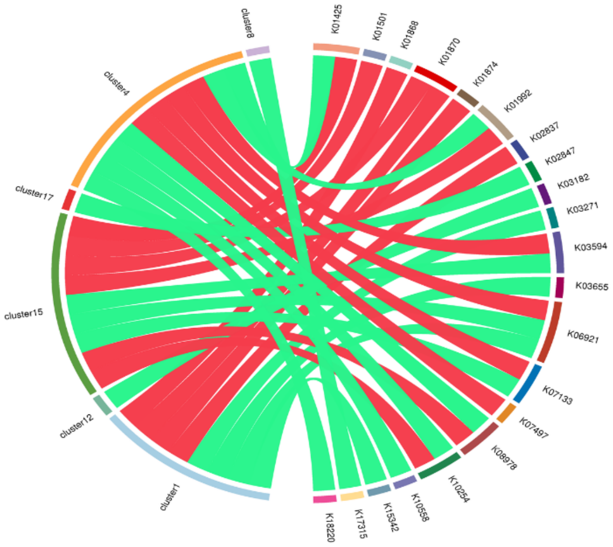

Figure S14. Integrated Analysis of Metagenomics and Metabolomics.Chord diagram of the correlation between metabolite clusters and microbial functions.

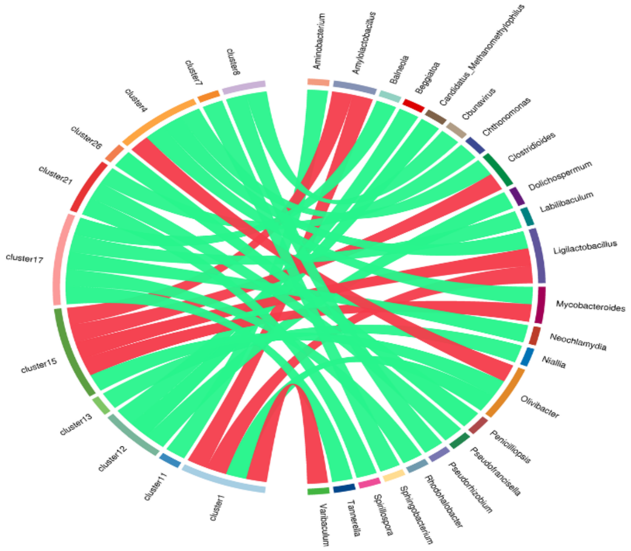

Figure S15. Integrated Analysis of Metagenomics and Metabolomics. Chord diagram of the correlation between metabolite clusters and microbial species.

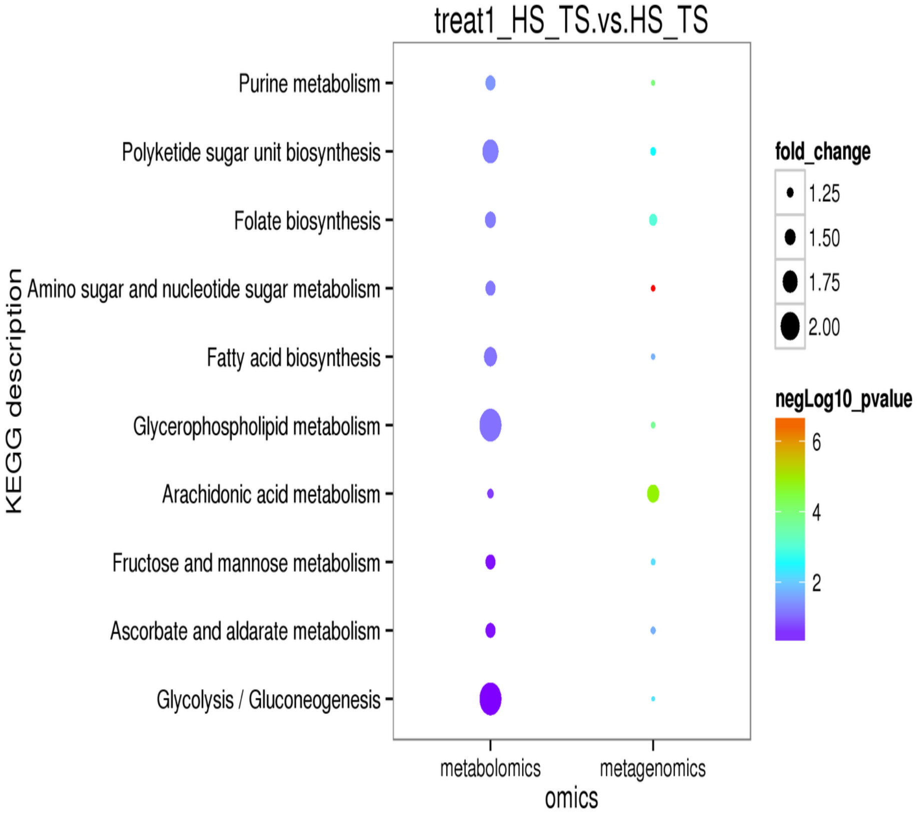

Figure S16. Integrated Analysis of Metagenomics and Metabolomics.Scatter plot of KEGG pathway analysis for differential metabolites and differential microbial functional genes.
